# The Ratio of Silicon to Chitin in Diatom Cell Walls Influences Their Resilience to Environmental Stress

**DOI:** 10.34133/research.1452

**Published:** 2026-10-07

**Authors:** Mengyuan Hao, Runyu You, Zhouyi Jiang, Linwei Liu, Lu Huang, Thomas Mock, Naikun Shen, Junrong Liang

**Affiliations:** ^1^ School of Life Sciences, Key Laboratory of the Coastal and Wetland Ecosystems, Ministry of Education, Xiamen Key Laboratory of Plant Genetics, Xiamen University, Xiamen, China.; ^2^ School of Environmental Sciences, University of East Anglia, Norwich, UK.; ^3^ College of Environmental Science and Engineering, Ocean University of China, Qingdao, China.; ^4^ Guangxi Key Laboratory for Polysaccharide Materials and Modifications, School of Marine Sciences and Biotechnology, Guangxi Minzu University, Nanning, China.

## Abstract

The diatom cell wall is a critically protective barrier against biotic and abiotic stress, yet the mechanisms by which its individual components confer resilience remain elusive. Using the model diatom *Thalassiosira pseudonana*, we show that resting cells chemically remodel their cell wall by increasing the ratio of silicon to chitin. Our integrative approach, based on a combination of transcriptomics, cell biology, and reverse genetics, reveals that the girdle-band-associated protein cingulin W2 (CinW2) directly regulates the biochemical cell wall composition, driving a “high silicon–low chitin” phenotype under abiotic and biotic stress. The regulation of the gene encoding CinW2 is controlled by an AP2-family transcription factor (AP2-1) that also modulates a chitinase, thereby orchestrating the compositional shift. This shift supporting a silicon-rich frustule provides enhanced structural reinforcement, substantially improving the resilience of *T. pseudonana* under biotic and abiotic stress. Tara Oceans metatranscriptome data suggest that genes involved in adjusting the ratio of silicon to chitin are widespread across diatom lineages and their expression appears to be responsive to changing environmental conditions in surface oceans. Although the CinW2-mediated regulatory mechanism has been identified only in the genus *Thalassiosira*, we have identified coregulated homologs in diverse diatom lineages. Collectively, our data therefore suggest that the molecular mechanism by which *T. pseudonana* modulates key cell wall components might represent a common strategy to cope with challenging environmental conditions.

## Introduction

The cell wall is a defining structural feature especially in plants, fungi, and bacteria, providing essential mechanical support, mediating environmental interactions, and acting as the primary barrier against abiotic and biotic stressors [1–4]. For photosynthetic organisms, the evolution of a protective yet adaptable cell walls has been central to their ecological success [5–7]. Among these, diatoms represent one of the most ecologically successful and diverse groups of photosynthetic organisms in aquatic systems such as the oceans [8,9]. Their dominance across dynamic marine environments is largely attributed to their intricate siliceous cell wall, known as the frustule [10–13]. This structure is presumed to function as a physical barrier, protecting cells from mechanical stress, grazing, viral entry, and ultraviolet radiation [10–13]. However, effectively coping with such a diverse array of stressors likely requires a degree of structural and biochemical plasticity in the cell wall to enable appropriate phenotypic responses. However, whether and how diatoms modify the plasticity of their frustules in response to specific stressors remains insufficiently studied, representing a critical gap in understanding their resilience.

The diatom frustule is a highly intricate, bipartite structure composed primarily of silica (SiO_2_), with opposing valves connected by girdle bands that form a hierarchical porous network spanning nano- and microscales [14]. Its protective capacity stems from a composite architecture that, while predominantly silicon based, also incorporates various minerals (e.g., C, N, O, P, S, Mg, Na, and Cl) and organic components [15,16]. Foundational studies, largely conducted in the model species *Thalassiosira pseudonana* (aka *Cyclotella nana* [17]), have identified key proteins associated with frustule formation. Silaffins (Sil1 to Sil4) are primarily associated with valve formation, while cingulins (e.g., CinW1 to CinW3 and CinY1 to CinY3) are girdle-band-specific proteins [18–21]. Recent functional studies have begun to elucidate the roles of silaffins: Sil3 appears to regulate cell size and macropore density, while Sil1/2 contribute primarily to macropore morphogenesis without affecting mesopore patterns [22]. However, functional in vivo studies on cingulins were limited prior to the present work. Long-chain polyamines participate in nanoscale silica polymerization in vitro, acting synergistically with frustule-associated proteins such as silaffins, silacidins, and cingulins [23,24]; nevertheless, in vitro activity does not constitute proof of in vivo function, and the precise roles of these components in frustule biogenesis remain to be established. Beyond these mineralization-associated proteins, the organic polymer chitin is another critical structural component, tightly integrated within the silica matrix. In the genus *Thalassiosira*, chitin localizes to the girdle bands and forms fibers extending from the fultoportulae of the valve [16,25] and has been proposed to serve as a scaffold guiding silica deposition during cell wall formation [16,25,26]. Interestingly, chitin production in the diatom frustule varies inversely with silicon availability. Silicon depletion enhances chitin production in *Cyclotella* species such as *Cyclotella cryptica* and *Cyclotella meneghiniana*, while prolonged silicic acid starvation up-regulates chitin synthase genes and promotes girdle-band localization of a chitin-binding lectin in *T. pseudonana* [25,27,28]. This compositional plasticity suggests that diatoms may adjust these essential frustule components to optimize resilience. However, the functional importance of the silicon-to-chitin ratio, particularly its direct role in determining the protective capacity of cell walls under stress, has remained elusive.

Diatoms encounter diverse environmental challenges in marine ecosystems, including both biotic and abiotic stressors. Among biotic stressors, chitinase-producing bacteria, which are naturally ubiquitous in aquatic environments, can directly target the chitin-containing structures of the diatom frustule [29,30], thereby serving as relevant models for natural microbial degradation pressure and offering a biological challenge to assess the protective role of frustule composition. In anthropogenically influenced coastal waters, diatoms may also be exposed to organic pollutants derived from fossil fuel combustion and industrial discharge [31,32]. Benzo[*a*]pyrene (BaP), a toxic polycyclic aromatic hydrocarbon, is one of those contaminants and has therefore been widely used as a model compound to assess pollutant-induced oxidative stress in phytoplankton [33,34]. Dissolved BaP concentrations have been reported to reach up to 4.80 μg/l in Daya Bay [35], 0.29 to 19.46 μg/l in the Gulf of Suez [36], and ~8.6 μg/l in an Indian river [37]. Importantly, BaP exposure has been linked to silicon metabolism: exposure of *T. pseudonana* to 36.45 μg/l BaP for 24 h down-regulated the silicon transporter 1 (SIT1), which is responsible for silicon uptake, suggesting that BaP exposure may influence biosilicification [33]. Together, these 2 distinct challenges provide useful experimental models for testing whether frustule composition influences susceptibility to abiotic and biotic stress. However, whether diatoms cope with these stressors through adaptive modification of their frustules and associated metabolism remains poorly understood.

To survive unfavorable conditions, many diatoms enter a dormant state, such as resting cells or spores [38,39]. Unlike spores or dinoflagellate cysts, which achieve enhanced resistance through obvious structural remodeling of the cell wall, diatom resting cells remain morphologically similar to vegetative cells [40,41]. This morphological similarity, combined with their hypometabolic nature, makes the biochemical fine-tuning of the frustule particularly critical for cellular protection and provides a unique opportunity to investigate how compositional adjustments, rather than structural remodeling, contribute to stress resilience. Using *T. pseudonana*, we previously characterized its resting cells by physiological and metabolic characterizations of energy depletion, intracellular acidification, chloroplast morphological alterations [42], and, during long-term survival, lipid remodeling and sulfur metabolism reprogramming [43]. Despite substantial intracellular adjustments during resting-cell formation, the siliceous frustule remains the primary barrier to protect the cells. However, not much is known about mechanisms underpinning the protective nature of cell walls in diatom resting cells. Thus, characterizing frustule plasticity in diatom resting cells offers a valuable perspective on how biochemical adjustments mediate adaptation to environmental (biotic and abiotic) stress.

To address this question, transcription factors (TFs) need to be considered as they likely play an important role in regulating the biochemical composition of the frustule during resting-cell formation. However, to date, the identification of regulatory components involved in the formation of diatom frustules is still in its infancy. Although diverse TF families, including myeloblastosis (MYB), basic leucine zipper (bZIP), basic helix–loop–helix (bHLH), AP2/ethylene responsive factor (AP2/ERF), and Cys2-His2 zinc finger (C2H2), have been suggested as regulators of cell-cycle progression and stress responses in diatoms [44–51], no individual TF from any of these families has been experimentally validated to be responsible for regulating the chemical composition of the diatom frustule at least to the best of our knowledge.

In this study, using the model diatom *T. pseudonana*, we investigated how essential frustule components, specifically silicon and chitin, contribute to the protective capacity of the cell wall under abiotic and biotic stress. By integrating physicochemical, transcriptomic, and reverse-genetics approaches, we demonstrate that the girdle-band-specific protein cingulin W2 (CinW2) and the AP2/ERF-family TF AP2-1 regulate stress resilience by increasing the silicon-to-chitin ratio in the frustule. This adaptive strategy appears to be conserved across diverse diatom lineages, providing novel insights into the mechanism underpinning diatom resilience to abiotic and biotic environmental stress.

## Results

### The physicochemical nature of resting-cell frustules

Compared to vegetative cells, the resting cells of *T. pseudonana* exhibited a markedly reduced cell size, with the mean valve diameter decreasing from 5.10 ± 0.57 to 3.31 ± 0.32 μm and the cell volume shrinking to approximately one-third (23.78 ± 4.91 μm^3^) (Fig. 1A to D). The silicon content per unit cell surface area, determined by the molybdenum blue method, was significantly elevated in resting cells (Fig. 1E). This increase was accompanied by a higher abundance of silanol groups (Si–OH), as revealed by attenuated total reflection Fourier transform infrared spectroscopy (ATR-FTIR) and x-ray photoelectron spectroscopy (XPS) analyses of whole-cell surfaces (Fig. 1F and G and Table S1). This elevated Si–OH signal was retained in isolated, cleaned frustules from resting cells (Table S2), suggesting that the increase is associated with the frustule silica itself rather than an increase in soluble intracellular silicon pools. Resting cells also exhibited a higher surface potential (Fig. 1H), along with reductions in Barrett–Joyner–Halenda pore diameter and total pore volume (Fig. 1C and Fig. S1). ATR-FTIR and XPS revealed a decrease in carbon content (Table S1) and a near-complete loss of C–N–H vibrational peaks (Fig. 1I). Given that chitin is a major organic component of the frustule, we quantified its content per unit cell surface area and observed a significant reduction in resting cells (Fig. 1J), a finding corroborated by high intelligent and sensitive structured illumination microscopy (HIS-SIM) and flow cytometry of calcofluor white (CFW)-stained cells (Fig. 1K and L). Consequently, the silicon-to-chitin ratio in resting-cell frustules increased (Fig. 1M). To further localize this compositional shift, we performed energy-dispersive x-ray spectroscopy (EDS) analysis on the girdle band, which revealed enrichment of silicon in resting cells (Fig. 1N and Fig. S3). Collectively, these data indicate that resting-cell frustules are enriched in silicon and weakly polymerized silicate (Si–OH) and depleted in chitin compared to vegetative cells.

**Fig. 1.**
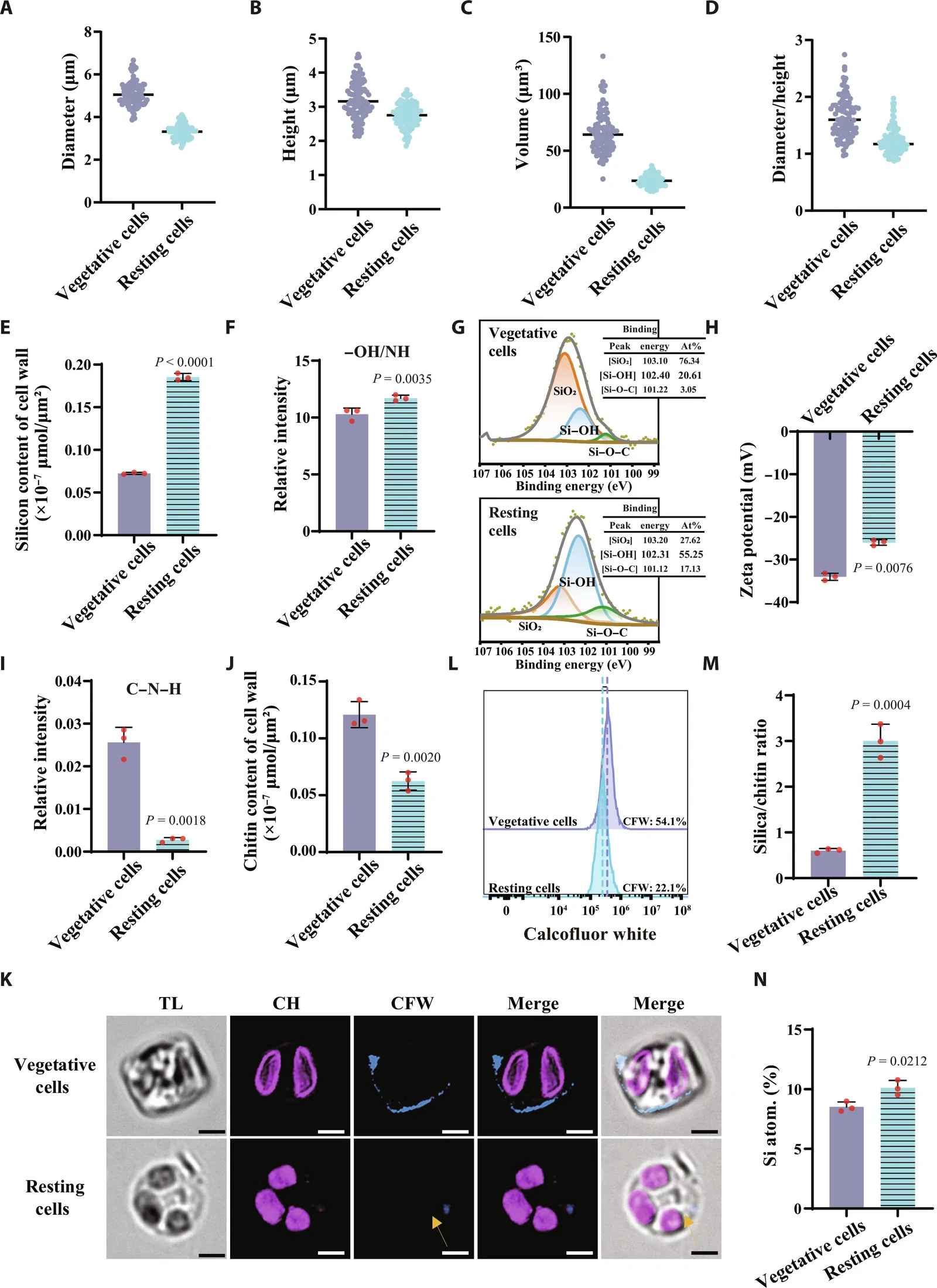
Physicochemical properties of the frustule in the resting cells of the diatom *Thalassiosira pseudonana*. (A to D) Cell diameter (A), cell height (B), cell volume (C), and diameter/height ratio (D) of vegetative and resting cells, measured from scanning electron microscopy (SEM) images (*n* = 100 cells from a single culture; horizontal lines indicate the mean). These data are descriptive and were not statistically tested. (E) Silicon (Si) content of the frustule per unit cell surface area in vegetative and resting cells. (F) Relative intensity of –OH/NH groups measured by attenuated total reflection Fourier transform infrared spectroscopy (ATR-FTIR). (G) X-ray photoelectron spectroscopy (XPS) Si 2p spectra of vegetative and resting cells, deconvolved into SiO_2_, Si–OH, and Si–O–C components; insets list binding energies and atomic percentages. (H) Surface zeta potential of vegetative and resting cells. (I) Relative intensity of C–N–H groups, measured by ATR-FTIR. (J) Chitin content of the frustule per unit cell surface area. (K) High intelligent and sensitive structured illumination microscopy (HIS-SIM) images of calcofluor white (CFW)-stained vegetative and resting cells. TL, transmitted light; CH, chlorophyll autofluorescence. Yellow arrows indicate CFW-positive chitin signals. Scale bars, 2 μm. (L) Flow cytometry of CFW-stained cells. (M) Silicon/chitin ratio of the frustules in vegetative and resting cells. (N) Si atomic percentage in girdle bands, determined by SEM–energy-dispersive x-ray spectroscopy (EDS). Error bars represent the mean ± SD of 3 biological replicates. The vegetative and resting cells were compared by 2-sided Student *t* tests.

### Transcriptome profiling of cell-wall-related genes

We previously analyzed the transcriptome of *T. pseudonana* resting cells and identified 4,142 differentially expressed genes [42]. This analysis, however, was conducted in the context of global dormancy physiology based on Kyoto Encyclopedia of Genes and Genomes enrichment analyses. In the present study, we reused this dataset for the analysis of genes involved in frustule formation. This new analysis identified the gene encoding CinW2 (*TpCinW2*, 7450309) as one of the most highly up-regulated transcripts globally (log_2_FC = 6.39), prompting us to investigate its potential role in the stress resilience of diatom frustules.

We selected 54 genes potentially associated with frustule formation based on previous studies. Among those, only 10 were significantly up-regulated in resting cells, whereas 28, including *TpSilicon transporters 2* and *3* (*TpSIT 2* and *TpSIT 3*) and *TpSilaffins 1* and *2* (*TpSil 1* and *TpSil 2*), were significantly down-regulated. Notably, *TpCinW2* was also the most highly expressed gene among the 54 selected candidates. Its protein product localizes to the girdle bands and the valve–girdle junction [20]. By contrast, the gene encoding silaffin (*TpSil4*, 7442038) was modestly up-regulated (log_2_FC = 1.25), and its encoded protein localizes to the valve and valve–girdle junction (Fig. S2A and Table S3) [19]. Several genes involved in the biosynthesis of long-chain polyamines were also significantly up-regulated, including 2 genes encoding S-adenosylmethionine decarboxylases (*TpSAM-decarboxylases 2* and *3*, 7447767 and 7452577; log_2_FC 3.34 to 6.83) and one gene encoding aminoacylase (*TpAminoacylase 1*, 7447450, log_2_FC = 2.17) (Fig. S2A and Table S4). Additionally, 2 genes associated with microtubules and actin organization were up-regulated (Fig. S2A and Table S4). Given that silicon was enriched at the girdle bands of resting cells (Fig. 1N and Fig. S3) and that CinW2 promotes silica polymerization in vitro [21], we prioritized it as the principal candidate driving the silicon-enriched phenotype, warranting further functional validation.

In contrast, genes involved in chitin metabolism exhibited the opposing trend. One gene that encodes chitinase protein (*TpChitinase*, 7450055) was significantly up-regulated (log_2_FC = 3.95), whereas enzymes required for chitin biosynthesis, particularly the gene encoding protein chitin synthase B (*TpChitin synthase B*, *7446358*), were down-regulated (log_2_FC −3.38 to −4.75) (Fig. S2A and Table S4). This coordinated expression pattern is consistent with the reduced chitin content observed in resting-cell frustules. Reverse transcription quantitative polymerase chain reaction (RT-qPCR) results largely corroborated the RNA sequencing (RNA-seq) data (Fig. S2B to D).

The coregulation of silicon- and chitin-associated genes may suggest the involvement of shared upstream *cis*-regulatory elements. To identify candidates, we examined the transcriptional landscape of TFs in resting cells. Among the 243 TFs-encoding genes annotated in the *T. pseudonana* genome, 92 were up-regulated and 68 were down-regulated. The up-regulated TFs mainly belonged to the heat shock factor (HSF), bZIP, MYB, AP2, and sigma families, whereas the down-regulated TFs were predominantly from the HSF, MYB, C2H2, C3H, and bZIP families (Table S5).

### The silicon and chitin content of genetically modified *T. pseudonana*

To test whether CinW2 is responsible for the altered frustule silicon content observed in resting cells, we generated *TpCinW2* overexpression (OE-CinW2) and CRISPR–Cas9-based knockout (KO-CinW2) cell lines (Figs. 2A and B and 3A and B). Sequencing of the edited locus confirmed that KO-CinW2-35 carries an out-of-frame deletion and represents a true null allele, while KO-CinW2-16 and KO-CinW2-23 contain in-frame deletions predicted to encode truncated proteins lacking 130 and 116 residues, respectively, of the 383-residue precursor (Fig. 3B). Thus, all 3 lines possess confirmed edits at the *TpCinW2* locus.

**Fig. 2.**
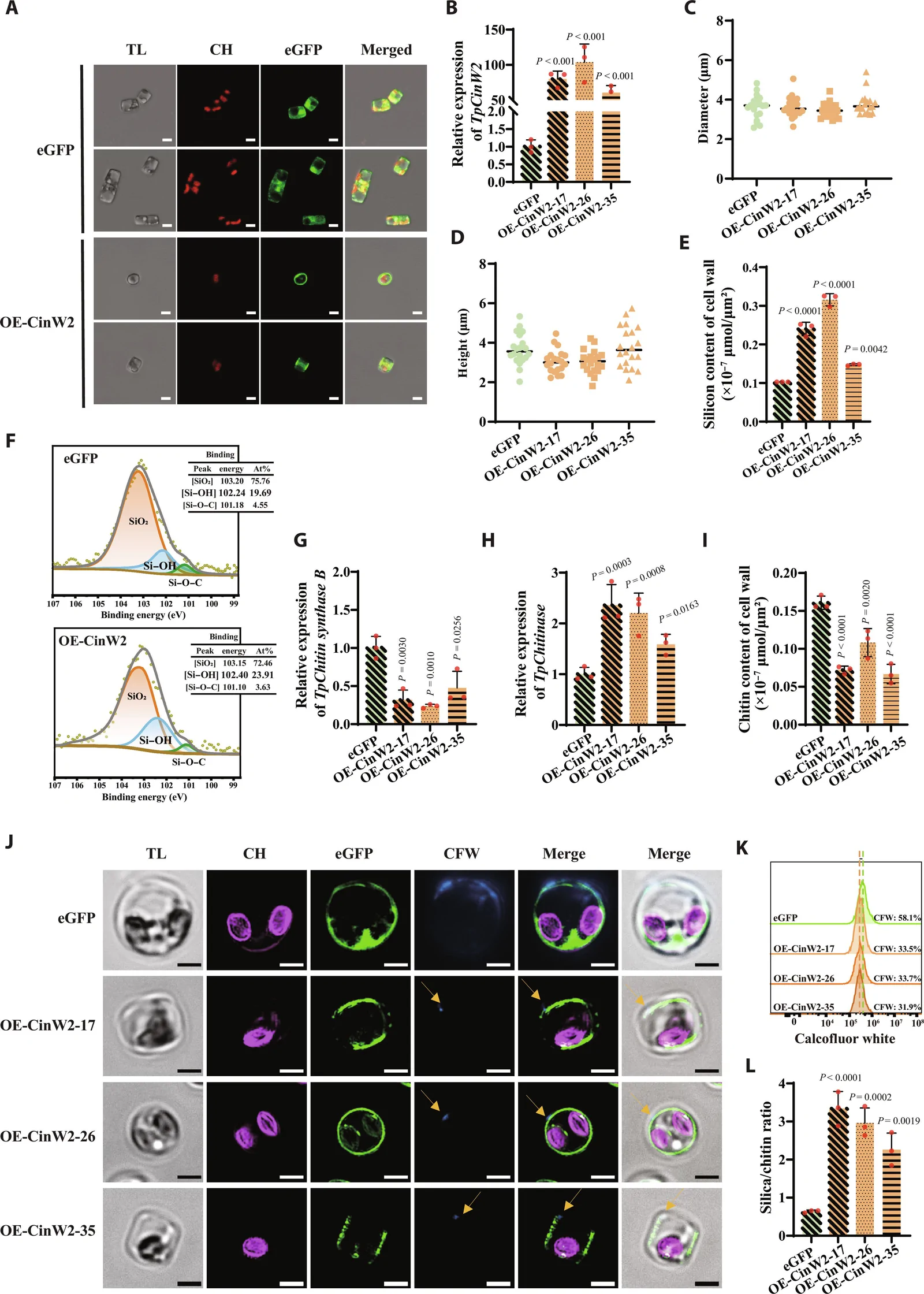
*TpCinW2* overexpression (OE) increases silicon content and decreases chitin in the frustule. (A) Representative fluorescence images showing the subcellular localization of CinW2-eGFP. Scale bars, 5 μm. CinW2, cingulin W2; eGFP, enhanced green fluorescent protein. (B) *TpCinW2* expression in OE-CinW2 lines determined by reverse transcription quantitative polymerase chain reaction (RT-qPCR). (C and D) Cell diameter (C) and cell height (D) of eGFP control and OE-CinW2 lines (*n* = 20 cells per line from a single culture; horizontal lines indicate the mean). (E) Silicon (Si) content of the frustule per unit cell surface area in eGFP and OE-CinW2 lines. (F) X-ray photoelectron spectroscopy (XPS) Si 2p spectra of the frustule from the eGFP and OE-CinW2 lines, deconvolved into SiO_2_, Si–OH, and Si–O–C components; insets list binding energies and atomic percentages. (G and H) Expression of the *TpChitin synthase B* gene (G) and *TpChitinase* gene (H) in OE-CinW2 lines determined by RT-qPCR. (I) Chitin content of the frustule per unit cell surface area. (J) High intelligent and sensitive structured illumination microscopy (HIS-SIM) images of calcofluor white (CFW)-stained eGFP and OE-CinW2 lines. TL, transmitted light; CH, chlorophyll autofluorescence. Yellow arrows indicate chitin. Scale bars, 2 μm. (K) Flow cytometry of CFW-stained cells. (L) Silicon/chitin ratio of frustules in the eGFP and OE-CinW2 lines. Error bars represent the mean ± SD of 3 biological replicates. Groups were compared by one-way analysis of variance (ANOVA) followed by Dunnett’s test against the eGFP control.

**Fig. 3.**
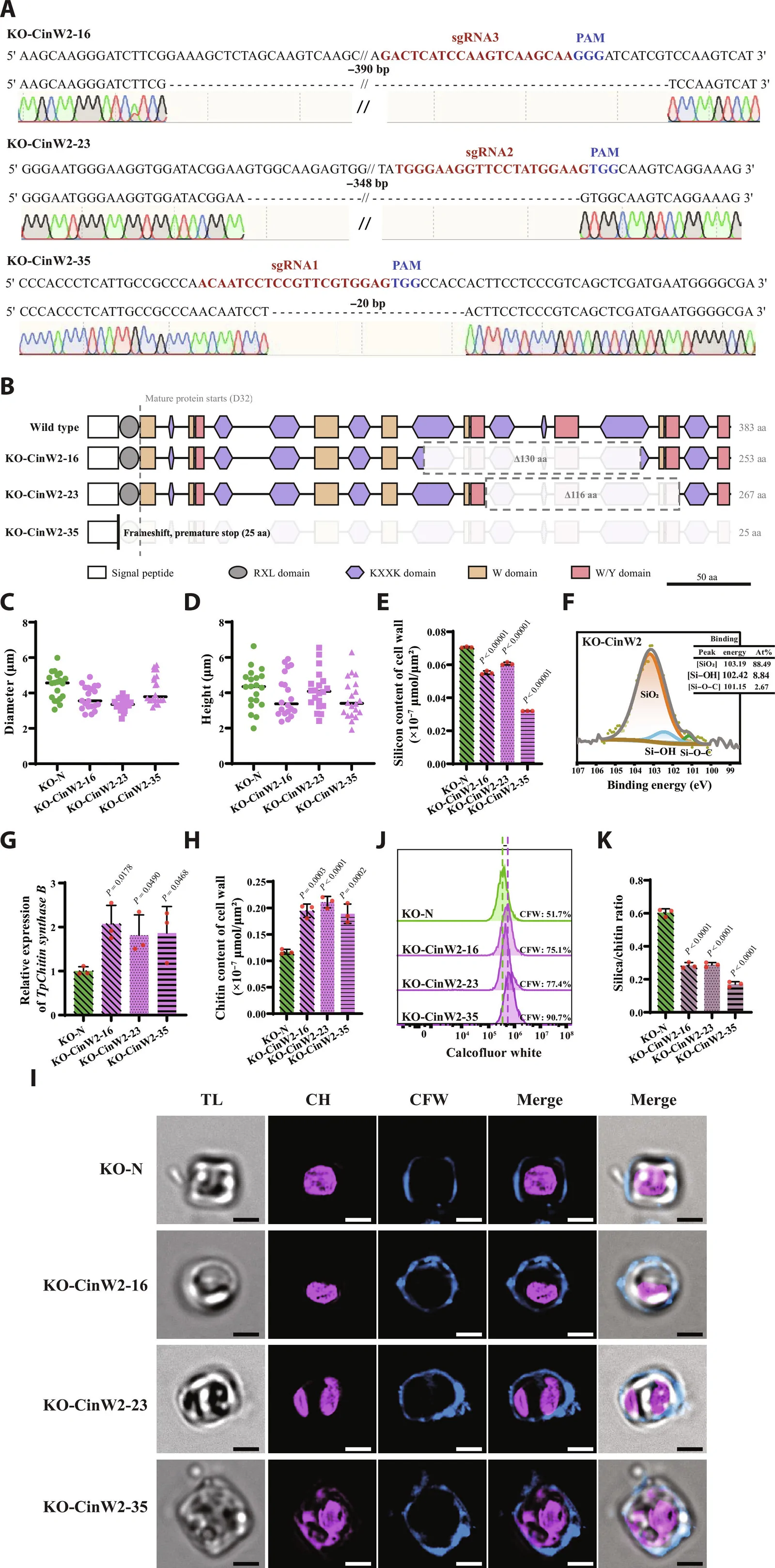
Genetic knockout (KO) and truncation of *TpCinW2* reduce silicon content and increase chitin in the frustule. (A) Sequence verification of 3 CRISPR–Cas9-edited KO-CinW2 lines. Single-guide RNA (sgRNA) target sites and protospacer adjacent motif (PAM) sequences are indicated. CinW2, cingulin W2. (B) Domain architecture of wild-type CinW2 and the predicted proteins from the 3 edited cell lines. Mutant proteins are drawn relative to wild-type residue numbering, and dashed boxes mark the deleted intervals. (C and D) Cell diameter (C) and cell height (D) of KO-N and KO-*CinW2* lines measured from scanning electron microscopy (SEM) images (*n* = 20 cells per line from a single culture; horizontal lines indicate the mean). KO-N is the nontargeting CRISPR–Cas9 control. (E) Silicon (Si) content of the frustule per unit cell surface area in KO-N and KO-CinW2 lines. (F) X-ray photoelectron spectroscopy (XPS) Si 2p spectra of frustules from KO-N and KO-CinW2 lines, deconvolved into SiO_2_, Si–OH, and Si–O–C components; insets list binding energies and atomic percentages. (G) Expression of the *TpChitin synthase B* gene in KO-CinW2 lines determined by reverse transcription quantitative polymerase chain reaction (RT-qPCR). (H) Chitin content of the frustule per unit cell surface area. (I) High intelligent and sensitive structured illumination microscopy (HIS-SIM) images of calcofluor white (CFW)-stained KO-N and KO-CinW2 lines. TL, transmitted light; CH, chlorophyll autofluorescence. Scale bars, 2 μm. (J) Flow cytometry of CFW-stained KO-N and KO-CinW2 lines. (K) Silicon/chitin ratio of the frustules in KO-N and KO-CinW2 lines. Error bars represent the mean ± SD of 3 biological replicates. Groups were compared by one-way analysis of variance (ANOVA) followed by Dunnett’s test against the KO-N control.

Genetic modifications did not appreciably affect cell size (Figs. 2C and D and 3C and D), allowing us to attribute subsequent changes in frustule composition to altered CinW2 function rather than the indirect effects of cell-size variation. Overexpression of the *TpCinW2* gene did not alter the subcellular localization of the protein, which remained at the girdle bands (Fig. 2A). OE-CinW2 lines exhibited a significantly elevated silicon content per unit cell surface area (Fig. 2E), accompanied by a higher proportion of Si–OH (Fig. 2F and Table S1). In contrast, KO-CinW2 lines showed a reduced silicon content and a lower proportion of Si–OH (Fig. 3E and F and Table S1). Concurrently, chitin metabolism genes showed reciprocal responses: in OE-CinW2 lines, the gene *TpChitin synthase B* was down-regulated, while *TpChitinase* was up-regulated (Fig. 2G and H); conversely, KO-CinW2 lines up-regulated *TpChitin synthase B* expression (Fig. 3G). As a consequence, cellular chitin content per unit cell surface area decreased in OE-CinW2 lines and increased in KO-CinW2 lines, as determined by CFW staining (Figs. 2I to K and 3H to J). Accordingly, the silicon-to-chitin ratio increased in OE-CinW2 lines but decreased in KO-CinW2 lines (Figs. 2L and 3K).

### Transcriptional regulation of *TpCinW2* and *TpChitinase*

To elucidate the regulatory basis of the chemical remodeling of frustules in *T. pseudonana*, we integrated promoter *cis*-element analysis with transcriptome data to identify candidate regulators. The promoter regions of both *TpCinW2* and chitin-related genes contained predicted binding motifs for AP2, MYB, bZIP, bHLH, and C2H2 TFs (Fig. 4A). In resting cells, up-regulated TFs mainly belonged to the HSF, bZIP, MYB, AP2, and sigma families (Table S5). Based on the overlap between promoter motif predictions and TF families up-regulated in resting cells, AP2, MYB, and bZIP were considered candidate families for identifying a TF.

**Fig. 4.**
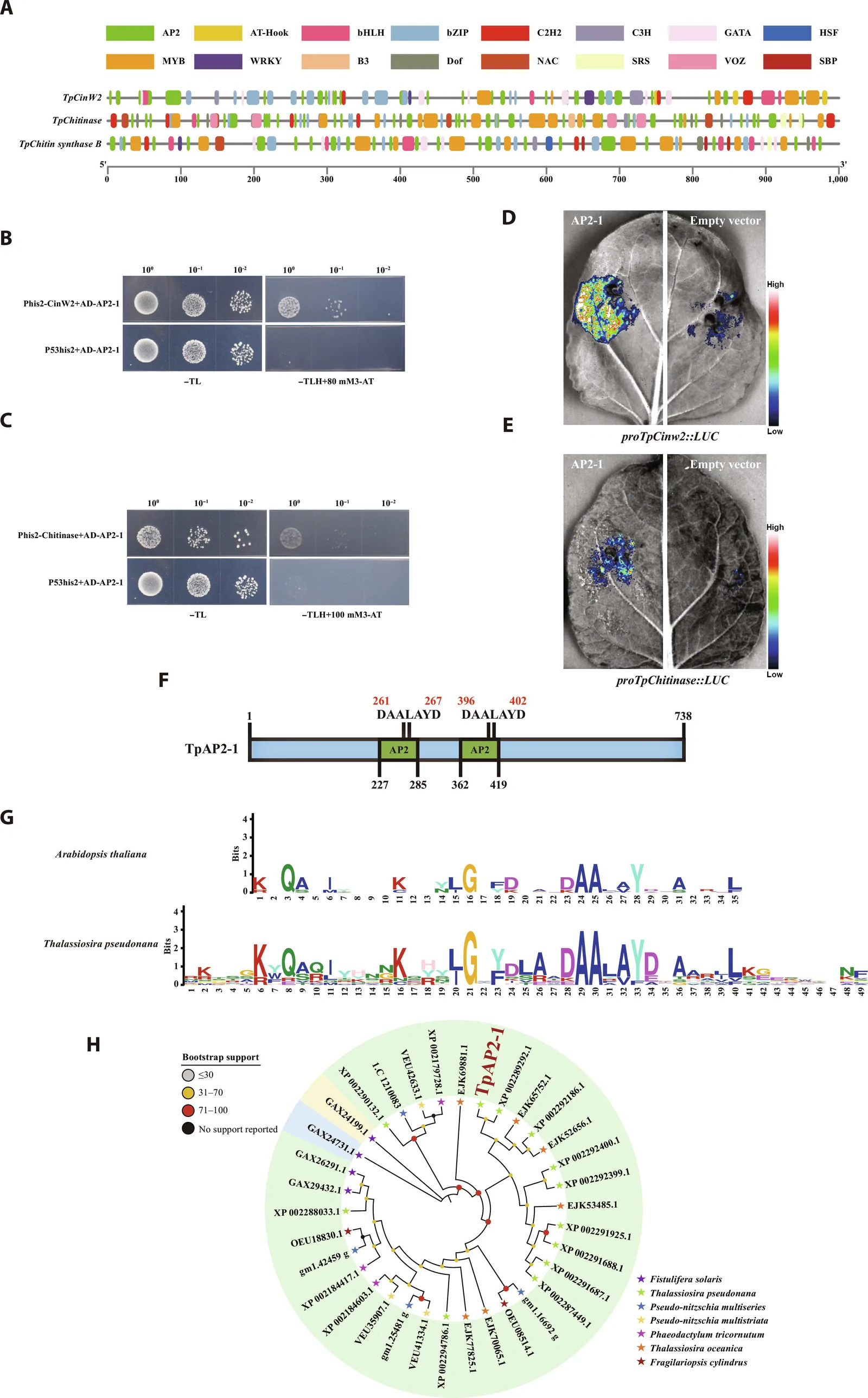
TpAP2-1 is a transcription factor that binds the promoters of *TpCinW2* and *TpChitinase*. (A) Predicted *cis*-acting elements in the promoter regions of *TpCinW2*, *TpChitinase*, and *TpChitin synthase B* identified using PlantPAN. (B to E) Yeast one-hybrid (Y1H) assays and dual-luciferase assays confirmed the direct interaction of TpAP2-1 with the promoter sequences of *TpCinW2* (B and D) and *TpChitinase* (C and E). (F) Predicted domain architecture of TpAP2-1. (G) Conserved motifs in TpAP2-1 and AP2 proteins from *Arabidopsis thaliana*, identified using MEME. (H) Phylogenetic analysis of AP2 homologs from 7 diatom species.

Given that many genes encoding MYB and bZIP were significantly down-regulated in the resting-cell transcriptome, we prioritized the AP2 family for further analysis. Among AP2-family members, 6 encoding genes were significantly up-regulated in resting cells (Table S5). We therefore selected one of these genes, which encodes AP2-1 (*TpAP2-1*, 7444978, log_2_FC = 3.10, Table S5), for subsequent study. We conducted yeast one-hybrid (Y1H) assays to test the interactions between AP2-1 and the promoters of *TpCinW2* and chitin-related genes. These assays demonstrated that AP2-1 directly binds to the promoters of *TpCinW2* and *TpChitinase*, but not to the promoter of *TpChitin synthase B* (Fig. 4B and C and Fig. S4A). We next performed dual-luciferase transient expression assays to validate the Y1H results. Effector constructs carrying pGreenII62SK-AP2-1 and reporter constructs containing CinW2::LUC, chitinase::LUC, or chitin synthase B::LUC were cotransformed into *Nicotiana benthamiana* leaf epidermal cells. Coexpression of AP2-1 with CinW2::LUC or chitinase::LUC substantially increased luciferase (LUC) activity relative to the empty-vector control, indicating that AP2-1 activates transcription from the *TpCinW2* and *TpChitinase* promoters (Fig. 4D and E). By contrast, no obvious change in LUC signal was observed for the chitin synthase B::LUC reporter (Fig. S4B). These results suggest that AP2-1 functions as a TF coordinating the expression of *TpCinW2* and *TpChitinase*.

Domain prediction using InterPro revealed that TpAP2-1 contains 2 canonical AP2 domains, spanning residues 227 to 285 and 362 to 419 (Fig. 4F). Moreover, MEME motif analysis revealed that the DAALAYD motif, which is also found in the AP2 domains of *Arabidopsis thaliana*, is present in the AP2 domains of TpAP2-1 (Fig. 4F and G), further supporting the conservation of its core features. To further assess its evolutionary relationships, we constructed a phylogenetic tree incorporating AP2 TFs from multiple diatom species based on conserved domain architecture and sequence similarity (Fig. 4H). AP2 homologs were resolved into 3 clades. TpAP2-1 was assigned to clade 3, which was the largest of the 3 clades in terms of the number of AP2 homologs. Within this clade, TpAP2-1 fell into a subclade that also contained 2 AP2 proteins from *T. pseudonana* and 2 AP2 proteins from *T. oceanica* (Fig. 4H), implying potential conservation of the associated regulatory role between these 2 species. Together, these findings suggest that AP2 proteins are broadly present across different diatom lineages and that they have retained conserved structural features despite substantial sequence divergence.

### The ratio of silicon to chitin in the diatom cell wall and its impact on mitigating abiotic and biotic stress

To test whether frustule remodeling enhances resistance to environmental stress, we exposed diatom cells to BaP, a widespread marine pollutant known to induce oxidative damage in phytoplankton [52–55]. Concentration–response assays (0.1 to 5 μg/l) revealed no detectable growth inhibition at 0.1 μg/l (Fig. S5A). However, at ≥1 μg/l, group-dependent differences emerged: OE-CinW2-35 maintained higher cell density and viability than the enhanced green fluorescent protein (eGFP) control, whereas KO-CinW2-23 showed growth suppression, reaching ~45% mortality at 5 μg/l (Fig. S5B to F). Based on these results, 5 μg/l was selected for subsequent experiments. At this concentration, resting cells maintained higher viability than vegetative cells, with cell abundance remaining stable in resting cells but decreased significantly in vegetative cells (Fig. 5A and E). OE-CinW2 lines outperformed the eGFP control and exhibited higher viability than vegetative cells (Fig. 5B, C, and F). In contrast, KO-CinW2 lines showed greater growth inhibition and cell death than the KO-N control (Fig. 5D and G). These results indicate that frustule composition, rather than dormant physiology alone, plays a key role in coping under BaP exposure. Scanning electron microscopy (SEM) further revealed that vegetative cells and KO-CinW2 lines predominantly displayed aberrant girdle bands (93.1% ± 3.8% of cells), whereas resting and OE-CinW2 lines largely preserved structural integrity, with only 13.9% ± 3.8% showing morphological damage (Fig. 5H to J). These results support an association between frustule integrity and BaP tolerance.

**Fig. 5.**
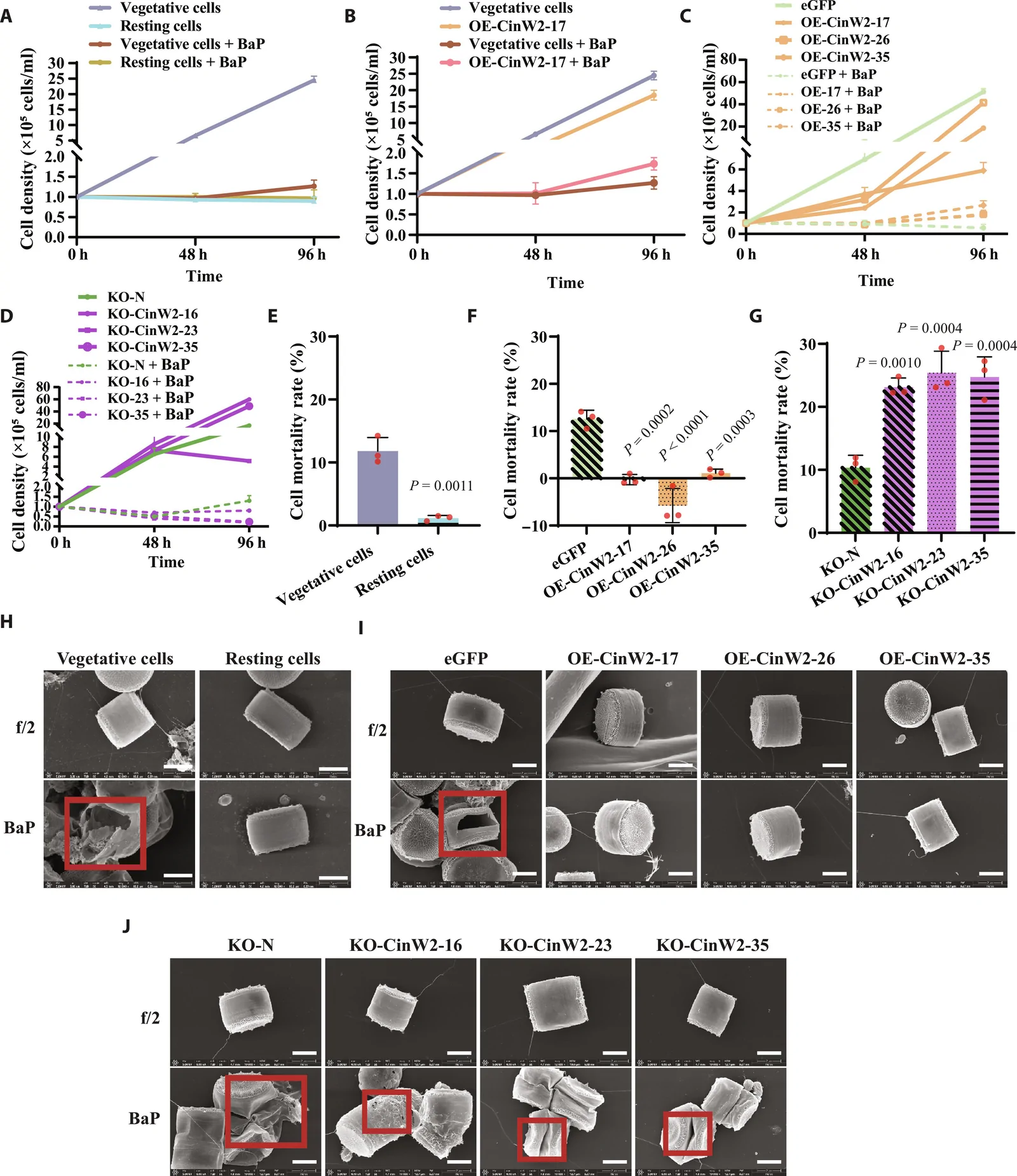
Resting cells and OE-CinW2 lines display enhanced tolerance to the marine pollutant benzo[*a*]pyrene (BaP). Transgenic lines were assayed as vegetative cells and compared with the corresponding transformation-round controls, namely, the enhanced green fluorescent protein (eGFP) line for the overexpression (OE) series and the nontargeting CRISPR–Cas9 line (KO-N) for the knockout series. (A to D) Cell density of vegetative and resting cells (A), the eGFP control and OE-*CinW2*-17 (B), all OE-*CinW2* lines and the eGFP control (C), and all KO-*CinW2* lines and KO-N (D) during 96 h of BaP exposure. (E to G) Cell mortality after 96 h of BaP exposure in vegetative and resting cells (E), OE-*CinW2* lines and the eGFP control (F), and KO-*CinW2* lines and KO-N (G). (H to J) Representative scanning electron microscopy (SEM) images of cells cultured in f/2 medium or exposed to BaP, showing vegetative and resting cells (H), the eGFP control and OE-*CinW2* lines (I), and KO-N and KO-*CinW2* lines (J). Red boxes indicate damaged or deformed girdle bands. Scale bars, 2 μm. Error bars represent the mean ± SD of 3 biological replicates. One-way analysis of variance (ANOVA) followed by Dunnett’s test was used to determine *P* values.

To assess whether frustule remodeling also confers resistance to biological stress, we performed co-culture experiments with 2 chitin-degrading bacteria, *Streptomyces beihaiensis* and *Chitinimonas prasina* LY03. These strains belong to distantly related lineages, allowing us to test whether the observed response is reproducible across different chitin degraders. During co-culture, resting cells and OE-CinW2 lines exhibited higher survival rates and minimal girdle-band deformities, whereas vegetative cells and KO-CinW2 lines showed reduced cell density in co-cultures and morphological deformities (Figs. S6 and S7). To distinguish whether the observed damage was caused by chitinase activity or by other biotic factors, we performed 2 additional controls. First, co-culture with heat-deactivated *S. beihaiensis* had no effect on the cell density of vegetative cells, OE-CinW2-17, or KO-CinW2-23 relative to that of bacteria-free controls (Fig. S8A), confirming that the damage requires metabolically active bacteria. Second, treatment with purified chitinase from *Streptomyces griseus* in the absence of bacteria reproduced the girdle-band damage in vegetative cells, whereas the frustules of treated resting cells remained morphologically intact (Fig. S8B). These results indicate that chitinase activity alone is sufficient to cause the observed damage. Taken together, these findings demonstrate that an elevated silicon-to-chitin ratio enhances frustule integrity and confers resistance to both chemical and biological stressors.

### Transcripts underpinning the ratio of silicon to chitin: From diverse diatoms to global oceanic datasets

To assess the ecological relevance of the observed cell wall plasticity, we examined the distribution and expression patterns of genes involved in silicon deposition and chitin metabolism using the Tara Oceans global metatranscriptome dataset. Using TpAP2-1 and the 54 potentially frustule-related proteins identified from our resting-cell transcriptome as queries, we retrieved homologs encoding key components of the silicon deposition machinery, including genes encoding SIT1 to SIT3, frustulins (*FRU1* to *FRU3*), silica matrix proteins (*SiMat3* to *SiMat5*), SiMat7 protein (aka silicanin 1, *Sin1*), silicalemma-associated proteins (*SAP1* and *SAP3*), and the TF AP2-1 (*AP2-1*). These genes were widely distributed across global ocean samples, with expression detected in diverse diatom-dominated communities (Fig. 6A to D and Fig. S9). This broad distribution suggests that the core molecular machinery for frustule silicification is conserved across diatom lineages and prevalent in natural marine environments.

**Fig. 6.**
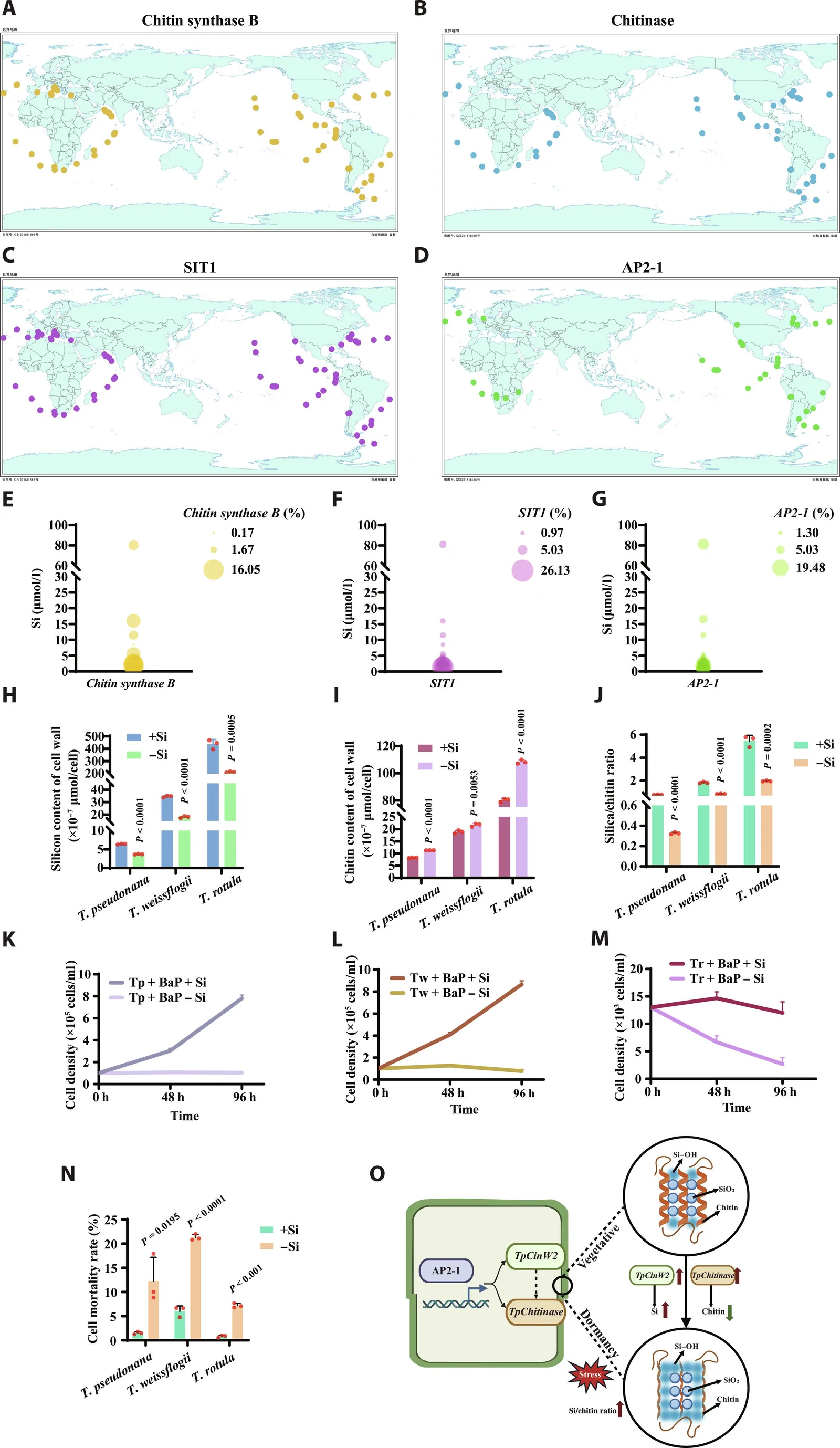
Adjusting the silicon/chitin ratio in the frustule can enhance stress resistance, which is applicable across *Thalassiosira* species. (A to D) Global distribution of homologs identified in the Tara Oceans metatranscriptomic dataset using chitin synthase B (A), chitinase (B), SIT1 (C), and AP2-1 (D) protein sequences as queries. (E to G) Relationships between ambient silicate concentration across sampling stations and the expression ratios of genes encoding chitin synthase B (E), SIT1 (F), and AP2-1 (G). (H to J) Frustule silicon content (H), chitin content (I), and silicon/chitin ratio (J) in *Thalassiosira pseudonana*, *Thalassiosira weissflogii*, and *Thalassiosira rotula* cultured under high-silicon (+Si) and silicon-depleted (−Si) conditions. (K to M) Cell density of *T. pseudonana* (K), *T. weissflogii* (L), and *T. rotula* (M) during 96 h of benzo[*a*]pyrene (BaP) exposure under +Si or −Si conditions. (N) Cell mortality after 96 h of BaP exposure. (O) Proposed model of AP2-1-mediated frustule remodeling in *T. pseudonana*. Tp, *T. pseudonana*; Tw, *T. weissflogii*; Tr, *T. rotula*. Error bars represent the mean ± SD of 3 biological replicates. All quantitative panels compare 2 independent groups (“Si^+^Chitin^−^” versus “Si^−^Chitin^+^”, nested within each species), and statistical analyses were performed using 2-sided Student *t* tests. This map was created based on the standard map downloaded from the Standard Map Service System of the Ministry of Natural Resources of the People’s Republic of China (review number: GS(2016)1666).

For chitin metabolism, we retrieved genes encoding chitin synthases (involved in chitin biosynthesis) and chitinases (involved in chitin degradation). Phylogenetic analysis of these genes across the Tara Oceans metatranscriptome dataset revealed their presence in multiple diatom lineages, including Thalassiosirophycidae, Coscinodiscophyceae, Fragilariophyceae, Mediophyceae, Cymatosiraceae, *Thalassiothrix*, and *Minutocellus* (Tables S6 and S7). In contrast, the genes encoding CinW2 in *T. pseudonana* was not retrieved from the Tara Oceans database. This is likely due to low sequence conservation of CinW2 outside the genus *Thalassiosira*, consistent with its lineage-restricted distribution inferred from phylogenetic analysis (Fig. 4H).

Despite the absence of CinW2-encoding genes in the global dataset, we observed marked negative correlations between silicate concentration and the expression ratios of genes encoding chitin synthase B and SIT1 (Fig. 6E and F and Tables S6 and S8). Given that increased silicate availability promotes silicon deposition and reduces chitin content in the frustule (as shown in Fig. 1), this negative correlation suggests that diatoms in natural environment can regulate chitin biosynthesis in response to silicate availability, which is closely related to silicon transport and deposition. Notably, the expression ratio of the genes encoding AP2-1, which we identified as an upstream regulator of *TpCinW2* and *TpChitinase* (Fig. 4B to E), also exhibited a negative correlation with ambient silicate concentration (Fig. 6G and Table S9), further supporting that the TF AP2 links silicate availability to the plasticity of diatom cell wall composition.

To experimentally test whether modulating the silicon-to-chitin ratio represents a conserved adaptive mechanism across the genus *Thalassiosira*, we examined 2 additional species, *Thalassiosira weissflogii* and *Thalassiosira rotula*. Both species adjusted their frustule composition in response to silicon availability. Under silicon-replete conditions, both species exhibited a higher silicon content and a lower chitin content (“Si^+^Chitin^−^”), whereas the opposite pattern (“Si^−^Chitin^+^”) was observed under silicon-depleted conditions (Fig. 6H and I). Consequently, the silicon-to-chitin ratio in the “Si^+^Chitin^−^” group was significantly higher than that in the “Si^−^Chitin^+^” group (Fig. 6J). When exposed to BaP, all “Si^+^Chitin^−^” groups exhibited significantly better growth and survival than their “Si^−^Chitin^+^” counterparts (Fig. 6K to N). These results demonstrate that the phenotypic plasticity of frustule composition is not restricted to *T. pseudonana* but may be broadly conserved across the genus. Nevertheless, whether CinW2 or AP2-1 homologs mediate this response in the other 2 species remains to be determined in future studies.

## Discussion

The resting cells of *T. pseudonana* are characterized by a global dormancy program involving metabolic suppression, energy depletion, and intracellular acidification, coupled with extensive membrane lipid remodeling (our previous studies [42,43]). Superimposed on this physiological quiescence, we uncovered a cell-wall-specific adaptive mechanism: AP2-1 transcriptionally activates the genes encoding CinW2 and a chitinase, driving a compositional shift toward silicon-enriched frustules. These 2 mechanisms, namely, systemic metabolic adjustment and local structural reinforcement, are likely to act synergistically to maximize cellular survival under biotic and abiotic stress. To explore how energy-depleted cells sustain active wall remodeling, we re-examined the temporal expression dynamics of the genes encoding CinW2, chitinase, and AP2-1 relative to adenosine triphosphate (ATP) depletion in our transcriptome time course. All 3 genes were up-regulated within the first 28 d of resting-cell induction and remained elevated throughout the 84-d induction period (Fig. S10A). By contrast, significant ATP depletion (to <30% of vegetative levels) occurred only after 56 d (Fig. S10B). Thus, the transcriptional activation of these genes precedes energy depletion. This temporal separation suggests that the transcriptional program for frustule remodeling is initiated early in resting-cell formation, when energy reserves are still sufficient, and may thus represent a prioritized investment in the primary protective barrier: the cell wall.

Diatoms can be distinguished from many other microalgae by their ability to construct a siliceous cell wall, although silica biomineralization also occurs in several other algal lineages such as the bolidophytes, haptophytes, silicoflagellates, and synurophytes [56–58]. However, the diatom frustule is thought to contribute substantially to the ecological success of diatoms by providing mechanical and chemical protection [10–13]. However, how diatoms modulate frustule plasticity in response to biotic and abiotic stressors remains poorly understood. Here, we provide evidence that diatoms enhance stress tolerance by increasing the silicon-to-chitin ratio in their frustule. This strategy was identified in the resting cells of the model diatom *T. pseudonana* and appears to be at least conserved within the genus *Thalassiosira*, with metatranscriptome data from the global surface ocean suggesting that it may also operate in other diatom lineages. The finding that a single TF, AP2-1, orchestrates the regulation of 2 functionally distinct genes, *TpCinW2* and *TpChitinase*, both of which influence frustule biochemistry, suggests that overarching regulatory networks must have evolved to integrate diverse metabolic processes in the maintenance and adjustment of diatom frustule composition. To the best of our knowledge, this is the first example of a regulatory unit coordinating genes with distinctly different functions toward the targeted biochemical modification of the diatom frustule. Interestingly, AP2-family TFs such as SHINE1/WAX INDUCER1 (SHN1/WIN1) are well-known in plants to regulate the cell wall structure [59]. This raises the possibility that AP2-family TFs in diatoms may serve analogous roles, and their prevalence in diatom genomes (Fig. 4H) makes them promising candidates for future studies aimed at elucidating the regulatory mechanisms underlying frustule architecture.

By remodeling the balance between silicon and chitin in the frustule, *T. pseudonana* achieves an optimal structural–functional trade-off under varying stress conditions, thereby enhancing its adaptability. Our analysis of the Tara Oceans data suggests that adjusting silicon and chitin contents possibly represents a widespread adaptive strategy across diverse diatoms if those identified gene expression changes match the biochemical processes as measured in the model *T. pseudonana*. Together, these findings expand our understanding of the adaptive mechanisms, including their regulatory components, underlying the ecological success of diatoms with respect to the role of their cell walls. We emphasize, however, that our mechanistic model is currently supported by functional evidence only in *T. pseudonana*; cross-species data and Tara Oceans correlations are suggestive of a widespread strategy but do not demonstrate conservation of the specific molecular mechanism. Future functional validation in additional diatom species will therefore be required to establish a concept of mechanistic conservation.

Usually, diatom silicification is a cell-cycle-dependent process based on silica (SiO_2_) deposition in vesicles (SDV) [60]. However, resting cells do not divide anymore. Thus, it is likely that silicification is decoupled from the cell cycle with consequences of how silicification takes place [42]. Our observation that the resting cells of *T. pseudonana* do not incorporate 2-(4-pyridyl)-5-((4-(2-dimethylaminoethylaminocarbamoyl)methoxy)phenyl)oxazole (PDMPO) [61] (Fig. S11) supports the hypothesis that canonical SDV-mediated silica biosynthesis does not occur during the formation of resting cells. Instead, the accumulation of silanol groups (Fig. 1F and G) points to an alternative, SDV-independent pathway for silicon assimilation during dormancy. Notably, Mayzel et al. [62] recently provided structural evidence for extracellular silica formation in diatoms, demonstrating that certain silicified structures can form outside the cytoplasm via a mechanism distinct from classical SDV-dependent silicification. This raises the possibility that the Si–OH-enriched silica observed in resting cells may similarly arise through an alternative, potentially extracellular pathway. Interestingly, our OE-CinW2 lines phenocopy the high silanol groups characteristic of resting cells, whereas the KO-CinW2 lines reduce the percentage of silanol groups (Figs. 2F and 3F). Given that recombinant CinW2 protein can promote silica polymerization in vitro [21], we propose a model where CinW2, localized to girdle bands, may facilitate the direct deposition or structural reorganization of silicic acid (Si(OH)_4_) from the external environment onto the existing frustule in resting cells. This potential extracellular silicification mechanism remains hypothetical and warrants further investigation, but it highlights the biochemical versatility of diatom frustule maturation even in the absence of an active cell cycle.

The marked reduction in cell size observed in resting cells is likely a direct consequence of chitin remodeling. Chitin forms an integral organic scaffold within the silica matrix, particularly in girdle bands [25]. The concerted up-regulation of chitinase, potentially acting extracellularly [63], and the down-regulation of chitin synthase would minimize the chitin content of the cell wall. In other biological systems, such as bacteria and plants, the removal or alteration of key structural wall polymers such as chitin influences cell wall curvature and mechanics, thus causing morphological changes [64,65]. By analogy, we speculate that the loss of chitinous support in diatom frustule may compromise the frustule’s ability to maintain its original architecture, potentially leading to a controlled inward contraction and the formation of smaller, denser, and more robust shells (Fig. S1). This hypothesis is consistent with observations in other systems [65–67], but direct evidence in diatoms is currently lacking and therefore warrants future investigation. Notably, this size reduction increases the surface-to-volume ratio, which would enhance nutrient uptake efficiency upon reentry into vegetative growth.

The inverse relationship between silicon and chitin content represents a fundamental trade-off in diatom frustule biology. Under silicon-replete conditions, a heavily silicified frustule provides optimal structural defense. Conversely, when silicon becomes limiting, diatoms compensate by up-regulating chitin synthesis to maintain cell wall integrity, which is a compensatory strategy analogous to cell wall remodeling observed in fungi [25,64,68]. This compositional shift from silicon-dominated to chitin-reinforced walls coincides with the transition from bloom initiation to bloom decline in diatom populations. During the exponential growth phase, ample silicon availability supports robust silicification, whereas under silicon depletion toward the late stage of blooms, increased chitin incorporation may serve as a survival mechanism. However, this adaptive shift incurs an ecological cost: chitin is a target for degradation by ubiquitous marine bacterial chitinases [69,70], rendering the frustule more susceptible to enzymatic attack. Compromised frustule integrity not only weakens physical protection but also heightens vulnerability to additional stressors and bacterial colonization [71,72], thereby accelerating population decline and contributing to the termination of diatom blooms. Subsequently, the lysis of compromised cells releases silicic acid back into the surrounding water, which can promote the formation of a resilient, high-silicon “seed bank” of resting cells. This process establishes a feedback loop that links cell wall composition to population dynamics and biogeochemical cycling, ultimately shaping diatom resilience and succession in marine ecosystems. However, we acknowledge that the proposed model remains theoretical and requires validation through time-series field surveys and direct chemical characterization of frustule composition in natural populations. Our correlative findings should therefore be interpreted as generating testable hypotheses rather than providing definitive evidence of ecological causality.

In conclusion, we have identified the first evidence that the ratio of silicon to chitin is orchestrated by at least one specific TF (AP2-1) from the AP2 family regulating expression of the genes encoding CinW2 and chitinase. This adaptive strategy appears to be at least conserved in the genus *Thalassiosira*. However, Tara Oceans metatranscriptomes hint at a broader and more conserved mechanism potentially existing in diverse diatom lineages underpinning resilience to biotic and abiotic stress (Fig. 6O). Thus, our work bridges molecular cell biology with ecology, showing how the regulation of a single structural protein, CinW2 in *T. pseudonana*, coordinated with chitin metabolism can influence a species’ resilience to abiotic and biotic stressors, thereby shaping survival under unfavorable conditions. Understanding such plastic responses is crucial for predicting how these ecologically vital phytoplankton will fare in a changing ocean subjected to multiple stressors, including pollution, nutrient shifts, and evolving microbial interactions.

## Materials and Methods

### Study design

The objective of this study was to systematically investigate the role of diatom frustule remodeling in enhancing stress resistance in *T. pseudonana* and to elucidate the molecular mechanisms underlying this process, with particular emphasis on the regulation and functional consequences of the silicon-to-chitin ratio. The study combined physiological, biochemical, molecular, genetic, microscopic, and bioinformatic approaches. Vegetative cells and resting cells induced by prolonged darkness at low temperature were used to compare frustule composition, surface chemistry, cellular morphology, and stress tolerance. Silicon and chitin contents were quantified using colorimetric assays, while frustule localization and structural features were examined by CFW staining, flow cytometry, superresolution microscopy, SEM, ATR-FTIR, XPS, and zeta potential analysis. To identify candidate regulators associated with frustule remodeling, previously published RNA-seq data were reanalyzed and selected genes were validated by RT-qPCR. The function of *TpCinW2* was further assessed using overexpression and CRISPR–Cas9 knockout lines in *T. pseudonana*, followed by measurements of frustule silicon and chitin contents, subcellular localization, and resistance to chemical and biological stressors. The regulatory relationship between AP2-1 and key frustule-related genes was evaluated using Y1H and dual-luciferase assays. Stress resistance was tested by exposing diatom cells to BaP or to the chitinase-producing bacteria *C. prasina* LY03 and *S. beihaiensis*, followed by analyses of cell density, viability, and frustule integrity. Additional comparative experiments using *T. weissflogii* and *T. rotula*, together with Tara Oceans-based homolog searches, were used to assess whether silicon- and chitin-related genes and frustule compositional patterns extend beyond the primary model species.

### Culture conditions and resting-cell induction

The centric diatom *T. pseudonana* (strain CCMP 1335) was used as the primary model. Additional experiments included *T. weissflogii* and *T. rotula* (strains obtained from Xiamen University). All strains were maintained axenically in f/2 medium at 20 °C under a 12-h:12-h light:dark cycle with an irradiance of 130 μmol photons m^−2^ s^−1^.

The resting cells of *T. pseudonana* were induced as previously described [42]. Briefly, late-exponential-phase vegetative cells were harvested, washed, and resuspended in fresh f/2 medium. Cultures were then wrapped in foil and incubated in complete darkness at 4 °C for 84 d. Cells displaying rounded, compact chloroplasts were identified as resting cells, a morphological feature validated by our previous study [42] using multiple objective criteria, including metabolic suppression (respiration reduced to ~21% of vegetative levels; *F*_v_/*F*_m_ < 10%), intracellular acidification (cytoplasmic pH ~ 6.0), and increased cytoplasmic and chloroplastic viscosity.

### Morphological measurements and cell surface area estimation

Measured cells were fixed, dehydrated through an ethanol series, critical-point dried, sputter-coated with gold, and imaged using a Hitachi SU8010 scanning electron microscope. For each cell state, 100 morphologically intact cells were randomly selected; for each transformant line, 20 cells were selected. Valve diameter (*D*) and the distance between the upper and lower valves (cell height, *H*) were measured using the ImageJ software (National Institutes of Health, USA). Cell volume was calculated from valve diameter and cell height using cylindrical geometric models, and cell surface area was estimated by approximating each cell as a cylinder: *S* = *π* × *D* × *H* + (*π* × *D*^2^)/2.

For each cell state or line, all measured cells originated from a single culture. Therefore, these data describe cell-to-cell variation within a culture rather than independent biological replicates and were used solely for descriptive characterization of cell-size distributions, without inferential statistical testing. Silica and chitin contents were normalized to the mean surface area of the corresponding cell state or line and are reported as micromoles/square micrometer.

### Frustule silicon and chitin content measurement

Frustule purification: Cells were grown in silicon-free f/2 medium in polyethylene flasks to the mid-exponential phase. A 100-ml aliquot (≈10^6^ cells ml^−1^) was centrifuged (3,220 × *g*, 10 min). The pellet was resuspended in 6 ml of extraction buffer (2% SDS, 100 mM EDTA pH 8.0, and 1 mM phenylmethylsulfonyl fluoride [PMSF]), incubated at 55 °C with shaking for 1 h, and centrifuged (3,220 × *g*, 2 min). The pellet was washed sequentially with 10 mM EDTA (pH 8.0, containing 1 mM PMSF) until the supernatant was colorless, followed by 1 wash with acetone and 4 washes with Milli-Q water. Purified frustules were finally resuspended in 1 ml of H_2_O.

Silicon quantification: A colorimetric molybdenum blue method was used. Briefly, 1 ml of purified frustule suspension was digested with 500 μl of 6 M NaOH at 95 °C for 30 min. A 50-μl aliquot was mixed with 150 μl of H_2_O, 100 μl of glacial acetic acid, 40 μl of buffer (1.5 M sodium acetate, pH 4.0), and 20 μl of ammonium molybdate reagent (10% w/v). After mixing, the solution was incubated at 60 °C in the dark for 10 min, cooled for 5 min, and then mixed with 100 μl of 20% (w/v) citric acid. Following a 20-min incubation at room temperature, 20 μl of a reducing agent (2% w/v ascorbic acid, 2% w/v sodium sulfite, and 0.05% w/v 1-amino-2-naphthol-4-sulfonic acid) was added. After a final 20-min incubation, absorbance was measured at 800 nm. A standard curve was prepared using sodium silicate.

Chitin quantification: Purified frustules were digested with 0.47 to 0.65 U ml^−1^ chitinase (from *S. griseus*, Sigma) in 50 mM potassium phosphate buffer (pH 6.5) containing 0.05% sodium azide and 1 mM PMSF for 3 h at 37 °C. The released *N*-acetylglucosamine was quantified using the Morgan–Elson assay. An aliquot of 200 μl of digest was mixed with 0.25 ml of 4% acetylacetone in 1.25 M Na_2_CO_3_, heated at 100 °C for 30 min, and cooled. Then, 2 ml of ethanol and 0.25 ml of Ehrlich’s reagent (1.6 g of *p*-dimethylaminobenzaldehyde in 30 ml of concentrated HCl and 30 ml of ethanol) were added, followed by incubation at 60 °C for 1 h. Samples were centrifuged (13,000 × *g*, 10 min), and the absorbance of the supernatant was measured at 530 nm. Chitin content was calculated from a standard curve of *N*-acetylglucosamine.

The per-cell silica or chitin content of each replicate was divided by a strain-level mean surface area determined from SEM measurements.

### ATR-FTIR analysis of diatom surface functional groups

Diatom cells were harvested by centrifugation at 3,500 × *g* for 5 min and washed 3 times with sterile seawater. Pellets were snap frozen at −20 °C for at least 12 h and subsequently freeze-dried in a lyophilizer (Venus 4.5 L; −55 °C) under 1-mbar vacuum until complete dehydration. Dried samples were analyzed using a Fourier transform infrared spectrometer (Thermo Nicolet iS 5) across a spectral range of 4,000 to 500 cm^−1^ with a resolution of 4 cm^−1^. Spectral acquisition, smoothing, calibration, and analysis were performed using the OMNIC software. The peak positions and integrated areas of characteristic functional groups were quantified. To enable comparisons among treatments, spectra were normalized to the amide I band, as relative absorbance intensity is proportional to functional group abundance. Mean relative peak intensities were calculated, and one-way analysis of variance (ANOVA) was used to evaluate significant differences among physiological states [73].

### XPS analysis of diatom frustule surface chemistry

XPS was used to characterize the chemical states of silicon at the sample surface (probing depths of 2 to 10 nm) [74,75]. Mid-exponential-phase vegetative cells and 4-month-old resting cells were harvested, freeze-dried, and mounted onto aluminum sample holders. XPS measurements were performed using a K-ALPHA spectrometer (Thermo VG Scientific) equipped with an Al Kα radiation source (1,486.6 eV) operated at 300 W. Spectra were acquired at an energy resolution of 0.5 eV. The chemical states of surface elements, including carbon (C 1s), oxygen (O 1s), nitrogen (N 1s), phosphorus (P 2p), silicon (Si 2p), sulfur (S 2p), magnesium (Mg 2p), sodium (Na 1s), and chlorine (Cl 2p), were analyzed. The C 1s peak at 284.8 eV was used for charge correction, and a linear background was subtracted prior to analysis. Spectral deconvolution and curve fitting were performed using the Origin 9.0 software [15].

### Zeta potential measurement of diatom cells

The zeta potential of diatom cells was measured using a nanoparticle size and zeta potential analyzer (Zetasizer Ultra, Malvern Panalytical). Approximately 10^6^ cells were harvested and resuspended in 800 μl of distilled water. The suspension was loaded into a zeta potential cell, and measurements were performed following the manufacturer’s instructions [76].

### EDS analysis by SEM–EDS

Cells were harvested by centrifugation (3,500 rpm, 8 min), washed 3 times with f/2 medium to remove residual debris and loosely associated extracellular impurities, and air-dried on a polished alumina (Al_2_O_3_) ceramic disc. Alumina was used in place of conventional glass or silicon supports, as both would contribute Si Kα counts indistinguishable from the diatom frustule signal. Samples were examined using field-emission SEM at an accelerating voltage of 10.0 kV and a working distance of 11.1 mm. X-ray spectra were collected with a detector at a magnification of 35,000×.

### RNA-seq and transcriptomic analysis

Gene expression analysis utilized a previously published RNA-seq dataset for *T. pseudonana* resting cells [42] (NCBI SRA: PRJNA1043413). Raw reads were mapped to the *T. pseudonana* genome (GCF_000149405.2) using HISAT2. Gene level read counts were obtained with HTSeq v0.9.1. Differential expression analysis was performed using DESeq2 (version 1.18.0) with an adjusted *P* value (*P*_adj_) <0.05 and a |log_2_ fold change| >1 as significance thresholds. Expression levels are reported as FPKM (fragments per kilobase of transcript per million mapped reads).

For several genes included in this analysis, particularly those involved in chitin metabolism, cytoskeletal organization, and polyamine biosynthesis, their specific roles in frustule formation versus other cellular processes remain to be established. Their inclusion here is based on known or suspected associations with cell-wall-related processes, and further functional characterization will be required to confirm their direct involvement in frustule biogenesis.

### Reverse transcription quantitative PCR

Total RNA was extracted using the Eastep Super Total RNA Extraction Kit (Promega). First-strand complementary DNA (cDNA) was synthesized from 1 μg of DNase-treated RNA using the PrimeScript RT reagent kit with gDNA Eraser (Takara). RT-qPCR was performed on a LightCycler 480 II system (Roche) using the GoTaq RT-qPCR System (Promega). An actin-like gene served as the endogenous control. Relative gene expression was calculated using the 2^−ΔΔCt^ method with GraphPad Prism 9.0. The primers used are listed in Table S10.

### Vector construction and genetic transformation

Overexpression vector: The full-length *TpCinW2* coding sequence was amplified from *T. pseudonana* cDNA using primers containing EcoRV and KpnI sites. The primers used are listed in Table S10. The PCR product and the recipient plasmid pTpNR-GFP/fcpNat(-KpnI) were digested and ligated to generate the overexpression construct pTpCinW2-GFP, which drives *TpCinW2* expression from the nitrate reductase (NR) promoter.

CRISPR–Cas9 knockout vectors: Three single-guide RNA (sgRNA) sequences targeting *TpCinW2* were designed using the Broad Institute’s CRISPR Design Tool. Each sgRNA was cloned into the pCas9 NR vector via Golden Gate assembly to generate knockout plasmids pCas9 NR-gRNA1/2/3 (Table S10).

Transformation: Plasmids were introduced into *T. pseudonana* cells using a biolistic particle delivery system (PDS-1000/He, Bio-Rad). Transformants were selected on solid f/2 medium containing 100 μg ml^−1^ nourseothricin (ClonNAT).

### Microscopy and imaging

Fluorescence and bright-field imaging of diatom cells and CFW staining of cells were performed using a superresolution microscope, termed HIS-SIM, provided by Guangzhou CSR Biotech Co. Ltd. HIS-SIM images were collected and analyzed as described previously [77]. Images were acquired using a 100×/1.5 NA oil immersion objective (Olympus). Briefly, eGFP was excited with a 488-nm laser and a 525/20 nm filter, chlorophyll autofluorescence was excited with a 638-nm laser and a 667/30 nm filter, and CFW was excited with a 405-nm laser and a 450/50 nm filter. To further remove image background and noise in HIS-SIM images, a customized AI denoise model was used. This model followed UNet and transformer architecture [78–80] and trained as a supervised way based on various HIS-SIM organelle images with different signal-to-noise ratios.

### Y1H assay

Y1H assays were used to assess the binding of AP2-1 to the promoters of *TpCinW2*, *TpChitinase*, and *TpChitin synthase B*. The promoter regions of *TpCinW2*, *TpChitinase*, and *TpChitin synthase B* were cloned into the EcoRI/SacI-digested pHis2 vector. The full-length *TpAP2-1* coding sequence was amplified and inserted into the pGADT7-Rec2 vector between EcoRI and SmaI. Yeast cells were grown on the selective dropout medium SD/−Trp−Leu/−His with different concentrations of 3-amino-1,2,4-triazole. The primers used are listed in Table S11.

### Dual-luciferase transient expression assay in tobacco leaves

Promoters of *TpCinW2*, *TpChitinase*, and *TpChitin synthase B* were cloned into pGreenII0800-LUC to generate reporter plasmids. The *TpAP2-1* coding sequence was cloned into pGreenII62SK-HA to generate the effector plasmid. The reporter plasmid and the effector plasmid were transformed into GV3101 (pSoup), and then the 2 kinds of *Agrobacterium tumefaciens* were simultaneously transferred to the *N. benthamiana* leaves for LUC detection. Approximately 10 min before signal detection, leaves were sprayed with 1 mM D-luciferin (cat. no. 7903, BioVision, San Francisco, CA, USA). LUC activity was then imaged using a cooled charge-coupled device on a Tanon 5200 chemiluminescence imaging system (Tanon Science & Technology Co., Ltd., Shanghai, China). The primers used are listed in Table S11.

### Stress assays

Chemical stress (BaP): Diatom cultures were initiated at a density of 2 × 10^4^ cells ml^−1^ and exposed to BaP at a final concentration of 5 μg/l (diluted from a 10 ppm methanol stock; final methanol concentration 0.05% v/v) or solvent control. Cell density and viability (assessed by propidium iodide staining) were assessed after 96 h. To evaluate the response across a broader environmentally relevant concentration range, a 96-h concentration–response experiment was additionally performed at 0, 0.1, 1, 2.5, 3.75, and 5 μg/l BaP.

Biological stress (co-culture with bacteria): *C. prasina* LY03 and *S. beihaiensis* were cultured to the stationary phase, washed, and resuspended in f/2 medium. Bacteria were added to diatom cultures (final bacterial concentration ~2% v/v) and co-cultured in the dark at 20 °C for 48 h. Algal cell density and frustule integrity (assessed by SEM) were evaluated after co-culture. As a control, parallel co-cultures were established with heat-killed *S. beihaiensis* (autoclaved at 121 °C for 20 min; absence of intact bacterial cells confirmed by light microscopy).

Enzymatic stress (chitinase treatment): To test whether chitinase activity alone reproduces the frustule damage observed during bacterial co-culture, vegetative and resting cells (initial density: 2 × 10^4^ cells ml^−1^) were incubated with chitinase from *S. griseus* (Sigma-Aldrich; 0.47 to 0.65 U ml^−1^) in 50 mM potassium phosphate buffer (pH 6.5) at 37 °C for 3 h. Frustule integrity was then examined by SEM as described above.

The *C. prasina* LY03 strain was obtained from Xiamen University. *S. beihaiensis* (strain MCCC 1K08064) was obtained from the Third Institute of Oceanography, Ministry of Natural Resources.

### Flow cytometry

For chitin quantification, CFW-stained cells (10,000 events per sample) were analyzed on a NovoCyte Quanteon flow cytometer (ACEA Biosciences) with a 405-nm laser. For viability assays, propidium iodide-stained cells were analyzed (excitation: 535 nm; emission: 617 nm). Data were processed using the FlowJo software.

### Bioinformatic analysis of Tara Oceans data

Protein sequences were used as queries to search the Marine Atlas of Tara Oceans Unigenes using BLASTP (*E*-value cutoff: 1 × 10^−10^). The searches were performed through the Ocean Gene Atlas server (v2.0; https://tara-oceans.mio.osupytheas.fr/ocean-gene-atlas/) [81,82]. Global distribution maps were generated using Python based on the longitude and latitude coordinates of sampling stations, and the base map was obtained from the Standard Map Service System of the Ministry of Natural Resources of the People’s Republic of China (http://bzdt.ch.mnr.gov.cn/), with review number GS(2016)1666, and no modifications were made to the base map.

### Statistical analysis

Data are presented as mean ± standard deviation of 3 independent biological replicates (*n* = 3) per group, with a single culture-level value per replicate. Before analysis of variance, normality was assessed using the Shapiro–Wilk test on within-group residuals, and homogeneity of variance using the Brown–Forsythe Levene test. Diagnostics were interpreted descriptively due to limited power at *n* = 3; where a potential departure was indicated, variance-robust or log-transformed sensitivity analyses confirmed that conclusions were unchanged. For comparisons among 3 or more groups, one-way ANOVA was used, followed by Dunnett’s test for post hoc comparisons (each transgenic line vs. its shared control). This controls the family-wise error rate for treatment-versus-control comparisons. Tukey’s honestly significant difference results for all pairwise contrasts are provided in Table S12. Two-way ANOVA was applied for factorial designs (cell type × stress; line × treatment), with simple effects examined following significant interactions using independent-samples *t* tests and Holm adjustment. Two-group comparisons were analyzed by 2-sided independent-samples *t* tests. Exact *P* values, effect sizes, and 95% confidence intervals are reported in Table S12. Analyses were performed in Python (SciPy, statsmodels); figures were prepared in GraphPad Prism 9.0. A *P* value <0.05 was considered statistically significant.

## Data Availability

The data supporting the findings of this study are available within the article and its Supplementary Materials.
