# Supplementary material for: The Ratio of Silicon to Chitin in Diatom Cell Walls Influences Their Resilience to Environmental Stress
Source: Research (Wash D C). 2026 Oct 7;9:1452. doi: 10.34133/research.1452 (PMC13643027; doi:10.34133/research.1452)
Supplement: Supplementary file 1 [file research.1452.f1.zip › Fig. S9-review number-260923.pdf]

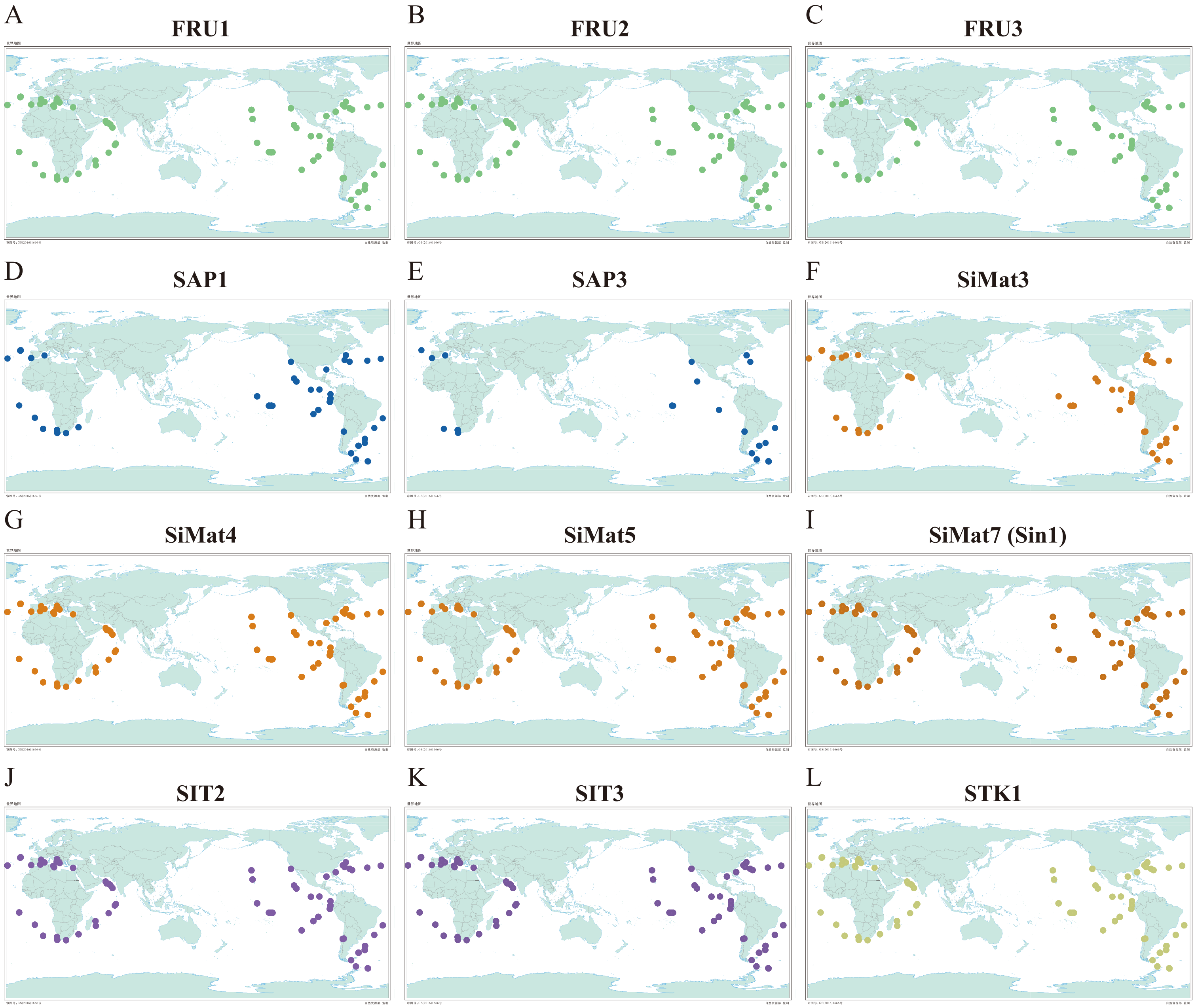
A
FRU1
B
FRU2
C
FRU3
D
SAP1
E
SAP3
F
SiMat3
G
SiMat4
H
SiMat5
I
SiMat7 (Sin1)
J
SIT2
K
SIT3
L
STK1
