# Supplementary material for: The Ratio of Silicon to Chitin in Diatom Cell Walls Influences Their Resilience to Environmental Stress
Source: Research (Wash D C). 2026 Oct 7;9:1452. doi: 10.34133/research.1452 (PMC13643027; doi:10.34133/research.1452)
Supplement: Supplementary file 1 [file research.1452.f1.zip › SUPPLEMENTARY MATERIALS.docx]

SUPPLEMENTARY MATERIALS

Figures S1 to S11

**Figure S1. Pore size distribution of purified frustules from vegetative and resting cells of *T. pseudonana*.**

**Figure S2. Transcriptional upregulation of genes encoding CinW2 and Chitinase in resting cells.** (A) RNA-seq analysis of resting cells. (B-D) Relative transcript levels of *TpCinW2* (B), *TpSil4* (C), *TpChitin synthase B* and *TpChitinase* (D) in vegetative and resting cells determined by RT-qPCR. Error bars represent the mean ± SD of three biological replicates. Relative expression values were log2-transformed, and vegetative and resting cells were compared by two-sided Student’s *t*-tests.

**Figure S3. Energy-dispersive X-ray spectroscopy (EDS) analysis of silicon distribution in the girdle bands of vegetative and resting cells.** (A-F) EDS analysis of girdle-band regions in vegetative cells (A-C) and resting cells (D-F). SEM images show representative regions analyzed in vegetative cells (A) and resting cells (D); corresponding EDS spectra (B, E) and Si elemental distribution maps (C, F). (G) Atomic percentage of silicon in the girdle bands of vegetative and resting cells. Data are presented as mean ± SD of three independent biological replicates (n = 3); individual replicate values are overlaid. Statistical significance was assessed using a two-sided Student’s *t*-test.

**Figure S4. Yeast one-hybrid (Y1H) analysis (A) and dual-luciferase reporter assay (B) confirmed that AP2-1 does not directly interact with the *TpChitin synthase B* promoter sequence.**

**Figure S5. Concentration-dependent effects of benzo[a]pyrene (BaP) on the growth and mortality of vegetative cells and *TpCinW2* transgenic lines.** (A-E) Cell density of vegetative cells, the *TpCinW2* overexpression line OE-CinW2-35 and its eGFP control, and the *TpCinW2* knockout line KO-CinW2-23 and its KO-N control during 96 h of exposure to BaP at final concentrations of 0.1 (A), 1 (B), 2.5 (C), 3.75 (D), and 5 μg/L (E). Cell densities were determined at 0, 48, and 96 h. Solid lines represent the corresponding untreated groups, whereas dashed lines represent BaP-treated groups. (F) Cell mortality rates of vegetative cells, eGFP, KO-N, OE-CinW2-35, and KO-CinW2-23 after 96 h of exposure to increasing concentrations of BaP (0.1-5 μg/L). Data are presented as mean ± SD from three independent biological replicates (n = 3). Strain × concentration interactions were assessed by two-way ANOVA (all *P* ≤ 0.003); each transgenic line was compared with its matched control at each concentration by t-tests with Holm adjustment (*P* < 0.05).

**Figure S6. Resting cells and OE-CinW2 strains show enhanced resistance to the chitinase-producing bacterium *Streptomyces beihaiensis*.** Transgenic lines were assayed as vegetative cells and compared with the corresponding transformation-round controls, namely the eGFP line for the overexpression series and the non-targeting CRISPR-Cas9 line (KO-N) for the knockout series. (A–C) Cell density of vegetative and resting cells (A), OE-CinW2 lines and the eGFP control (B), and KO-CinW2 lines and KO-N (C) after 48 h of co-culture with *S. beihaiensis*. (D–F) Cell mortality rates of vegetative and resting cells (D), OE-CinW2 lines and the eGFP control (E), and KO-CinW2 lines and KO-N (F) after 48 h of co-culture with *S. beihaiensis*. (G–I) Representative SEM images of cells cultured in f/2 medium or co-cultured with *S. beihaiensis*, showing vegetative and resting cells (G), OE-CinW2 lines and the eGFP control (H), and KO-CinW2 lines and KO-N (I). Red boxes indicate damaged or deformed girdle bands. Scale bars, 2 μm. Error bars represent the mean ± SD of three biological replicates. One-way ANOVA followed by Dunnett’s test was used to determine *P* values.

**Figure S7. Resting cells and OE-CinW2 strains show enhanced resistance to the chitinase-producing bacterium *Chitinimonas prasina* LY03.** Transgenic lines were assayed as vegetative cells and compared with the corresponding transformation-round controls, namely the eGFP line for the overexpression series and the non-targeting CRISPR-Cas9 line (KO-N) for the knockout series. (A–C) Cell density of vegetative and resting cells (A), OE-CinW2 lines and the eGFP control (B), and KO-CinW2 lines and KO-N (C) after 48 h of co-culture with LY03. (D–F) Cell mortality rates of vegetative and resting cells (D), OE-CinW2 lines and the eGFP control (E), and KO-CinW2 lines and KO-N (F) after 48 h of co-culture with LY03. (G–I) Representative SEM images of cells cultured in f/2 medium or co-cultured with LY03, showing vegetative and resting cells (G), OE-CinW2 lines and the eGFP control (H), and KO-CinW2 lines and KO-N (I). Red boxes indicate damaged or deformed girdle bands. Scale bars, 2 μm. Error bars represent the mean ± SD of three biological replicates. One-way ANOVA followed by Dunnett’s test was used to determine *P* values.

**Figure S8. Effects of chitinase and heat-inactivated *S. beihaiensis* on vegetative and resting cells.** (A) Cell density of vegetative cells, OE-CinW2-17, and KO-CinW2-23 with or without heat-inactivated S. beihaiensis during 48 h of cultivation. (B) SEM images of vegetative and resting cells after chitinase treatment. Error bars represent the mean ± SD of three biological replicates.

**Figure S9. Tara Oceans metatranscriptome analyses showed that proteins associated with silicification are widely distributed across global ocean environments.** (A-C) The Frustulins proteins, FRU1 (A), FRU2 (B) and FRU3 (C). (D, E) The Silicalemma-associated proteins, SAP1 (D) and SAP3 (E). (F-I) The Silica matrix proteins, SiMat3-5 and SiMat7 (aka Sin1). (J, K) The Silicate transporters proteins, SIT2 (J) and SIT3 (K). (L) The Serine/threonine protein kinase, STK1 (L). This map was created based on the standard map downloaded from the Standard Map Service System of the Ministry of Natural Resources of the People's Republic of China (review number: GS(2016)1666).

**Figure S10. Temporal dynamics of frustule-remodeling gene expression and cellular ATP content during resting-cell induction.** (A) Temporal expression patterns of the genes encoding CinW2, Chitinase, AP2-1, and Chitin synthase B during 84 days of resting cell induction. (B) Temporal changes in cellular ATP content during resting-cell induction, reproduced from Wang et al. (2024). Asterisks indicate significant differences relative to day 0 (P < 0.05). Panel B is reproduced from Wang et al. (2024).

**Figure S11. PDMPO staining reveals different patterns of newly deposited silica in vegetative cells (A) and resting cells (B).** Scale bars, 5 μm.

Tables S1 to S12

Table S1. X-ray photoelectron spectroscopy (XPS) analysis of frustule surface composition in vegetative cells, resting cells, and transgenic lines of *Thalassiosira pseudonana*.

| Element | Core-level | Assignment | Vegetative Cells | | | Resting Cells | | | OE-CinW2 | | | KO-CinW2 | | |
| --- | --- | --- | --- | --- | --- | --- | --- | --- | --- | --- | --- | --- | --- | --- |
|  |  |  | AP(%) | BE(eV) | CD(%) | AP(%) | BE(eV) | CD(%) | AP(%) | BE(eV) | CD(%) | AP(%) | BE(eV) | CD(%) |
| C | 1s | **C**-(C, H) | 63.34 | 284.80 | 68.83 | 38.22 | 284.80 | 67.93 | 64.39 | 284.80 | 78.85 | 39.62 | 284.80 | 70.42 |
|  |  | **C**-(O, N) |  | 286.32 | 24.22 |  | 286.37 | 22.50 |  | 286.61 | 12.64 |  | 286.55 | 21.13 |
|  |  | O-**C**-O/**C**=O  /**C**OOR |  | 288.64 | 6.95 |  | 288.50 | 9.57 |  | 288.50 | 8.51 |  | 288.35 | 8.45 |
| N | 1s | **N**_nonpr_ | 3.82 | 399.90 | 89.29 | 3.50 | 399.90 | 76.92 | 2.91 | 399.94 | 81.96 | 2.79 | 399.97 | 86.21 |
|  |  | **N**_pr_ |  | 402.10 | 10.71 |  | 402.14 | 6.93 |  | 402.10 | 18.04 |  | 402.25 | 13.79 |
|  |  | **N**-Si |  | - |  |  | 398.01 | 16.15 |  | - |  |  | - | - |
| O | 1s | **O-**Si | 20.74 | 532.60 | 64.52 | 30.12 | 532.23 | 77.48 | 22.69 | 532.70 | 68.49 | 38.40 | 532.76 | 64.10 |
|  |  | C-**O**-C/**O**=C |  | 531.54 | 35.48 |  | 531.34 | 22.52 |  | 531.54 | 31.51 |  | 531.68 | 35.9 |
| Si | 2p | **Si**O_2_ | 2.77 | 103.10 | 76.34 | 2.93 | 103.20 | 27.62 | 2.04 | 103.15 | 72.46 | 1.28 | 103.19 | 88.49 |
|  |  | **Si**-OH |  | 102.40 | **20.61** |  | 102.31 | **55.25** |  | 102.40 | **23.91** |  | 102.42 | **8.84** |
|  |  | **Si**-O-C/O-**Si**-C |  | 101.22 | 3.05 |  | 101.12 | 17.13 |  | 101.10 | 3.63 |  | 101.15 | 2.67 |
| Mg | 2p | **Mg**(OH)_2_ | 2.23 | 50.05 |  | 5.11 | 50.35 |  | 2.73 | 50.01 |  | 0.56 | 50.35 |  |
| S | 2p | **S**O_4_ | 1.39 | 168.49 |  | 2.25 | 168.90 |  | 1.17 | 168.36 |  | 0.68 | 167.83 |  |
| P | 2p |  | 0.27 | 132.94 |  | 0.26 | 132.89 |  | 0.19 | 133.85 |  | 0.33 | 133.59 |  |
| Na | 1s |  | 0.92 | 1071.08 |  | 5.74 | 1071.18 |  | 0.61 | 1070.92 |  | 7.89 | 1071.00 |  |
| Cl | 2p |  | 4.39 | 198.40 |  | 11.87 | 198.77 |  | 3.28 | 198.39 |  | 8.43 | 198.25 |  |

Notes: AP: atomic percentage; BE: binding energy; CD: component distribution, which refers to the proportion of each component peak. The XPS signals reported as “O-C-O / C=O / COOR,” “C-O-C / O=C,” and “Si-O-C / O-Si-C” reflect binding energy overlaps between chemically similar configurations. Definitive assignment requires orthogonal validation and should be interpreted with caution.

Table S2. X-ray photoelectron spectroscopy (XPS) analysis of frustule surface composition in vegetative cells, resting cells, and transgenic lines of *Thalassiosira pseudonana* (clean frustules).

| Element | Core-level | Assignment | Vegetative Cells | | | Resting Cells | | |
| --- | --- | --- | --- | --- | --- | --- | --- | --- |
|  |  |  | AP(%) | BE(eV) | CD(%) | AP(%) | BE(eV) | CD(%) |
| C | 1s | C-(C, H) | 54.84 | 284.8 | 69.93 | 45.06 | 284.8 | 54.05 |
|  |  | C-(O, N) |  | 286.5 | 23.78 |  | 286.4 | 28.11 |
|  |  | O-C-O/C=O |  |  |  |  |  |  |
| /COOR |  | 288.9 | 6.29 |  | 288.5 | 17.84 |  |  |
| N | 1s | Nnonpr | 3.45 | 399.9 | 84.75 | 6.46 | 399.9 | 84.75 |
|  |  | Npr |  | 402.0 | 8.47 |  | 402.1 | 12.71 |
|  |  | N-Si |  | 398.6 | 6.78 |  | 398.0 | 2.54 |
| O | 1s | O-Si | 28.95 | 532.5 | 90.91 | 36.23 | 532.3 | 86.96 |
|  |  | C-O-C/O=C |  | 531.0 | 9.09 |  | 531.4 | 13.04 |
| Si | 2p | SiO2 | 10.89 | 103.3 | 95.24 | 9.52 | 103.1 | 89.29 |
|  |  | Si-OH |  | 102.2 | **2.86** |  | 102.4 | **9.82** |
|  |  | Si-O-C/O-Si-C |  | 101.4 | 1.90 |  | 101.1 | 0.89 |
| Mg | 2p | Mg(OH)2 | 0.72 | 48.9 |  | 0.60 | 49.9 |  |
| S | 2p | SO4 | 0.06 | 167.9 |  | 0.28 | 167.6 |  |
| P | 2p |  | 0.21 | 132.7 |  | 0.56 | 132.9 |  |
| Na | 1s |  | 0.74 | 1071.0 |  | 1.19 | 1070.9 |  |

Notes: AP: atomic percentage; BE: binding energy; CD: component distribution, which refers to the proportion of each component peak. The XPS signals reported as “O-C-O / C=O / COOR,” “C-O-C / O=C,” and “Si-O-C / O-Si-C” reflect binding energy overlaps between chemically similar configurations. Definitive assignment requires orthogonal validation and should be interpreted with caution.

**Table S3. Biosilicification-related genes that are differentially regulated in vegetative cells and resting cells.**

| gene ID | annotation | log2FoldChange(Day 84 (Resting cells)vsVegetative cells) | pval(Day 84 (Resting cells)vsVegetative cells) | significant(Day 84 (Resting cells)vsVegetative cells) |
| --- | --- | --- | --- | --- |
| 7450309 | CinW2 | 6.3928 | 1.51E-216 | UP |
| 7443064 | Silacidin(anti sense) | 1.5633 | 2.53E-17 | UP |
| 7442038 | SIL4; silaffin protein | 1.2489 | 9.43E-10 | UP |
| 7448451 | FRU1; hypothetical protein | 0.96728 | 0.38588 | FALSE |
| 7450490 | ODC1; ornithine decarboxylase | 0.53055 | 0.047825 | FALSE |
| 7444773 | p150 | 0.23981 | 0.18064 | FALSE |
| 7452009 | SiMat2 | 0.089226 | 0.84813 | FALSE |
| 7451656 | SiMat5 | 0.046069 | 0.76726 | FALSE |
| 7452147 | SiMat1/CinY4 | -0.04897 | 0.91987 | FALSE |
| 7451979 | CinY2 | -0.35082 | 0.46753 | FALSE |
| 7445519 | SIL2; silaffin protein | -0.47155 | 0.091072 | FALSE |
| 7451986 | CinY3 | -0.4898 | 0.082843 | FALSE |
| 7449457 | SAP3 | -0.51032 | 0.091079 | FALSE |
| 7446109 | STK1 | -0.51756 | 0.076158 | FALSE |
| 7450065 | SIT1; silicic acid transporter, silicon transport | -0.54592 | 0.026906 | FALSE |
| 7445194 | FRU2; hypothetical protein | -0.54923 | 0.010179 | FALSE |
| 7452012 | CinY1 | -0.68088 | 0.35584 | FALSE |
| 7450503 | SiMat7/Silicanin1 | -1.2774 | 1.39E-14 | DOWN |
| 7451621 | SiMat6 | -1.4251 | 2.31E-19 | DOWN |
| 7446153 | SiMat3 | -1.6322 | 9.02E-19 | DOWN |
| 7451981 | SiMat4 | -1.6824 | 0.00056175 | DOWN |
| 7447140 | SIT2; silicic acid transporter, silicon transport | -1.8168 | 1.02E-21 | DOWN |
| 7444619 | SIT3; SIT3, silicic acid transporter-like protein | -1.9657 | 1.78E-15 | DOWN |
| 7448554 | CinW3 | -1.9853 | 1.79E-34 | DOWN |
| 7442845 | SAP1 | -2.0669 | 4.26E-29 | DOWN |
| 7442998 | p150 like | -2.2968 | 1.08E-31 | DOWN |
| 7444399 | SIL1; silaffin protein | -2.8835 | 3.91E-73 | DOWN |
| 7443856 | FRU3; frustulin protein | -3.4379 | 8.37E-11 | DOWN |

**Table S4. Differentially regulated chitin-related genes and other genes potentially involved in biomineralization.**

| gene ID | annotation | log2FoldChange(Day 84 (Resting cells)vsVegetative cells) | pval(Day 84 (Resting cells)vsVegetative cells) | significant(Day 84 (Resting cells)vsVegetative cells) |
| --- | --- | --- | --- | --- |
| 7452577 | S-adenosylmethionine decarboxylasef | 6.8325 | 1.15E-40 | UP |
| 7447876 | regulating microtubule | 4.7986 | 1.06E-78 | UP |
| 7449122 | S-adenosylmethionine decarboxylase | 4.2601 | 1.66E-57 | UP |
| 7450055 | Chitinase | 3.948 | 2.59E-32 | UP |
| 7447767 | S-adenosylmethionine decarboxylase | 3.3416 | 3.83E-37 | UP |
| 7447450 | aminoacylase 1 | 2.1682 | 7.75E-22 | UP |
| 7450632 | Chitinase | 1.3839 | 0.37728 | FALSE |
| 7450357 | actin organization gene | 1.002 | 1.57E-07 | UP |
| 7449000 | Arg1 arginase 1 | -0.077452 | 0.73241 | FALSE |
| 7448939 | regulating microtubule | -1.0156 | 2.96E-06 | DOWN |
| 7445014 | meckelin | -1.1115 | 0.010836 | DOWN |
| 7446807 | Chitinase | -1.1195 | 0.0017171 | DOWN |
| 7442307 | S-adenosylmethionine decarboxylase | -1.8443 | 3.27E-18 | DOWN |
| 7444567 | gamma-tubulin complex component 3 | -1.8497 | 6.26E-12 | DOWN |
| 7446867 | Chitinase | -2.8525 | 1.89E-40 | DOWN |
| 7447910 | chitin synthase | -3.3264 | 1.97E-71 | DOWN |
| 7446358 | chitin synthase B | -3.3757 | 2.37E-20 | DOWN |
| 7443476 | Chitinase | -3.4202 | 7.14E-71 | DOWN |
| 7443061 | Chitinase | -3.7736 | 1.46E-51 | DOWN |
| 7449554 | Chitinase | -3.8674 | 7.19E-63 | DOWN |
| 7450610 | chitin synthase | -4.3236 | 1.13E-51 | DOWN |
| 7446045 | Chitinase | -4.5077 | 2.12E-19 | DOWN |
| 7450611 | chitin synthase | -4.745 | 4.24E-64 | DOWN |
| 7446214 | Chitinase | -6.1198 | 3.33E-30 | DOWN |
| 7443293 | glycoside hydrolase | -6.8112 | 8.75E-158 | DOWN |
| 7447934 | Chitinase | -9.2416 | 1.04E-32 | DOWN |

**Table S5. Expression pattern of transcription factors.**

| gene_ID | week 12 log2 fold change | annotation | week 12 p value |
| --- | --- | --- | --- |
| 7451764 | 1.4984 | AP2-EREBP1 | 5.4512E-14 |
| 7445004 | -2.0235 | AP2-EREBP10 | 3.3775E-10 |
| 7444978 | 3.1049 | AP2-EREBP11 | 1.8956E-19 |
| 7449272 | 3.1879 | AP2-EREBP12_CCHH20 | 0.16053 |
| 7452500 | -1.2185 | AP2-EREBP2 | 7.5661E-06 |
| 7447788 | 2.8667 | AP2-EREBP3 | 0.25129 |
| 7452442 | 2.311 | AP2-EREBP4 | 5.2117E-22 |
| 7453348 | -1.5168 | AP2-EREBP5 | 0.027289 |
| 7452393 | -0.69381 | AP2-EREBP6 | 0.16297 |
| 7452396 | 0.8481 | AP2-EREBP7 | 0.0010471 |
| 7443432 | -1.8727 | AP2-EREBP8 | 0.0077155 |
| 7445003 | 4.474 | AP2-EREBP9 | 5.8365E-72 |
| 7446149 | -0.024526 | aureochrome1 | 1 |
| 7453029 | 0.89604 | aureochrome2 | 0.01097 |
| 7445354 | -0.83399 | aureochrome3 | 0.098847 |
| 7451934 | 0.32304 | aureochrome4 | 0.14019 |
| 7448073 | 0.75827 | bHLH1_PAS | 0.00007662 |
| 7443138 | 4.6257 | bHLH2_PAS | 1.8429E-96 |
| 7448245 | 0.70227 | bHLH3 | 0.0060012 |
| 7453287 | 1.1776 | bHLH7_PAS | 7.8626E-09 |
| 7442095 | 6.106 | bHLH8_PAS | 3.7985E-87 |
| 7451117 | 0.58105 | bZIP10 | 0.090103 |
| 7446760 | -3.5612 | bZIP11 | 3.2463E-15 |
| 7452327 | 1.2816 | bZIP12 | 1.1455E-10 |
| 7443011 | -2.4788 | bZIP13 | 1.1179E-07 |
| 7445906 | 2.4241 | bZIP14 | 5.3934E-31 |
| 7443783 | 4.4654 | bZIP15 | 1.823E-43 |
| 7449547 | -1.9705 | bZIP17 | 1.5256E-13 |
| 7449064 | 2.8594 | bZIP18 | 1.714E-16 |
| 7449572 | 3.7048 | bZIP22 | 1.109E-52 |
| 7442493 | 0.67279 | bZIP24a | 0.00075996 |
| 7452717 | 0.54324 | bZIP24b | 0.045512 |
| 7452716 | 2.5846 | bZIP25a | 7.4168E-22 |
| 7446289 | -2.7993 | bZIP25b | 8.386E-10 |
| 7445842 | 11.672 | bZIP5_PAS | 0 |
| 7452027 | 2.0771 | bZIP6_PAS | 8.7669E-13 |
| 7450821 | 1.1145 | bZIP7a/PAS | 0.00011152 |
| 7451626 | 5.2638 | bZIP7b/PAS | 2.9952E-156 |
| 7450802 | -2.1735 | bZIP8 | 5.636E-11 |
| 7450730 | -0.10861 | bZIP9 | 0.81538 |
| 7445083 | -0.7892 | CBF/NF1a | 0.024487 |
| 7450703 | -2.1948 | CBF/NF1b | 5.5086E-08 |
| 7444877 | -1.2154 | CBF/NF2 | 0.000004431 |
| 7447822 | -0.7307 | CBF/NF3 | 0.0027839 |
| 7451648 | -0.30033 | CBF/NF4 | 0.51101 |
| 7445292 | 0.66361 | CBF/NF5 | 0.034508 |
| 7444478 | -1.3426 | CCCH1 | 0.07224 |
| 7449220 | 5.5961 | CCCH10 | 8.7004E-166 |
| 7442874 | 1.1703 | CCCH11 | 1.7544E-07 |
| 7446332 | -1.8232 | CCCH12 | 0.00013898 |
| 7451710 | -0.6385 | CCCH2 | 0.0088338 |
| 7442881 | -0.79682 | CCCH21 | 0.24258 |
| 7452931 | -2.1434 | CCCH22 | 1.6756E-08 |
| 7443880 | 0.38336 | CCCH23 | 0.18094 |
| 7451675 | -0.85279 | CCCH24 | 0.16374 |
| 7446740 | -3.3977 | CCCH25 | 2.0855E-15 |
| 7446401 | 2.0036 | CCCH26 | 4.4058E-21 |
| 7449856 | -1.1058 | CCCH27_RING95 | 0.0074823 |
| 7447262 | 4.8972 | CCCH3 | 2.0205E-134 |
| 7443845 | 0.32748 | CCCH4 | 0.46916 |
| 7448537 | -5.3526 | CCCH5 | 3.5844E-31 |
| 7442133 | -2.0283 | CCCH6 | 1.675E-21 |
| 7442598 | 0.10061 | CCCH7_RING62 | 0.7214 |
| 7452678 | -0.99051 | CCCH8 | 0.099199 |
| 7444529 | 0.23336 | CCCH9 | 0.2606 |
| 7448537 | -5.3526 | CCH5 | 3.5844E-31 |
| 7442133 | -2.0283 | CCH6 | 1.675E-21 |
| 7442598 | 0.10061 | CCH7 | 0.7214 |
| 7452678 | -0.99051 | CCH8 | 0.099199 |
| 7444529 | 0.23336 | CCH9 | 0.2606 |
| 7443996 | 1.214 | CCHH1_Myb2R10 | 0.000092289 |
| 7443837 | 0.97942 | CCHH10 | 0.00098307 |
| 7445051 | 0.40033 | CCHH11 | 0.23882 |
| 7449917 | -2.7303 | CCHH12 | 1.8103E-09 |
| 7451520 | -2.7678 | CCHH2 | 9.2829E-33 |
| 7445698 | 1.0656 | CCHH21 | 4.5873E-06 |
| 7444489 | -2.4543 | CCHH22 | 1.7343E-18 |
| 7452368 | -0.4206 | CCHH23 | 0.20134 |
| 7441919 | -3.4251 | CCHH24 | 1.0668E-11 |
| 7442023 | -0.91717 | CCHH25 | 0.1854 |
| 7447551 | -1.3886 | CCHH26 | 1.8832E-06 |
| 7444146 | -1.4403 | CCHH27 | 0.0080729 |
| 7453331 | 0.94504 | CCHH28 | 0.00074285 |
| 7443748 | -1.5376 | CCHH3 | 2.2878E-08 |
| 7445334 | -3.4904 | CCHH4 | 0.00029524 |
| 7442241 | 0.98291 | cdc5 | 0.018039 |
| 7450974 | -0.57651 | CSF1 | 0.10045 |
| 7446797 | 1.4898 | CSF2 | 0.091732 |
| 7447471 | 0.05204 | CSF3 | 0.89521 |
| 7449424 | -2.1383 | CSF4 | 2.6974E-09 |
| 7451732 | -2.0183 | CSF5 | 4.2188E-11 |
| 7447009 | -2.0692 | CXC1 | 1.4761E-08 |
| 7453385 | 0.023378 | CXC2 | 0.95 |
| 7445534 | 0.85505 | CXC3 | 0.012575 |
| 7446510 | -1.8656 | CXC4 | 0.027083 |
| 7442601 | 0.63588 | CXC6 | 0.37233 |
| 7446689 | 1.084 | E2F-DP1a_SET1a | 0.014336 |
| 7444159 | 1.1624 | E2F-DP2 | 0.27658 |
| 7452739 | -0.16585 | E2F-DP3 | 0.48138 |
| 7442780 | 0.20475 | fungal_TRF1a | 0.38287 |
| 7450908 | -0.61749 | fungal_TRF1b | 0.024328 |
| 7451804 | 2.3535 | fungal_TRF2 | 1.3631E-28 |
| 7451805 | -0.45369 | fungal_TRF3 | 0.057549 |
| 7447841 | 0.34796 | Hox1 | 0.15632 |
| 7452487 | -0.54856 | Hox2 | 0.010315 |
| 7445892 | 1.9342 | Hox3 | 2.3717E-17 |
| 7446829 | -1.9343 | Hox5a | 0.34864 |
| 7450086 | 1.1828 | Hox6a | 6.1018E-09 |
| 7452372 | -2.7606 | Hox6b | 2.1789E-13 |
| 7443821 | 0.27477 | Hox7 | 0.88909 |
| 7446663 | 2.7687 | Hox8 | 6.8728E-17 |
| 7451040 | 3.9569 | HSF_1.1e | 1.5819E-91 |
| 7442085 | -2.3446 | HSF_1.2a | 1.4257E-40 |
| 7452640 | 2.8654 | HSF_1.2b | 4.9618E-46 |
| 7450793 | -0.13838 | HSF_1.2c | 0.4981 |
| 7449059 | -0.078664 | HSF_1.3d | 0.77002 |
| 7444093 | -2.3027 | HSF_1.3e | 1.023E-13 |
| 7449189 | 4.8962 | HSF_1.3f | 1.7696E-105 |
| 7448962 | 0.29596 | HSF_1.3g | 0.65667 |
| 7444101 | 1.2317 | HSF_1.3h | 2.7261E-08 |
| 7445825 | 5.5416 | HSF_1.3i | 3.2176E-128 |
| 7446802 | 6.6815 | HSF_1.3k | 5.9683E-94 |
| 7442413 | 0.80868 | HSF_1.4b | 0.0046982 |
| 7453271 | 0.5026 | HSF_1.4c | 0.062177 |
| 7449518 | 6.9411 | HSF_1a | 5.4219E-35 |
| 7442477 | -0.30584 | HSF_1b | 0.42522 |
| 7452986 | 1.198 | HSF_1c | 7.4025E-07 |
| 7453044 | 1.1724 | HSF_1d | 0.00051524 |
| 7445882 | -1.6614 | HSF_1e | 6.7307E-09 |
| 7445883 | -1.2533 | HSF_1f | 0.00001622 |
| 7448762 | 2.5669 | HSF_2.2a | 5.2572E-18 |
| 7451942 | 1.219 | HSF_2.2c | 0.0005617 |
| 7443763 | -2.0804 | HSF_2.2e | 9.3785E-11 |
| 7444014 | -3.1721 | HSF_2.3a | 2.6823E-57 |
| 7443423 | 4.3626 | HSF_2.3b | 0.28597 |
| 7451115 | -1.6133 | HSF_2.3c | 0.000010654 |
| 7448660 | 1.4693 | HSF_2.4a | 3.0097E-09 |
| 7442936 | 0.80557 | HSF_2.4b | 0.0122 |
| 7442181 | 1.4553 | HSF_2.4c | 0.013819 |
| 7442166 | -3.0784 | HSF_2.4d | 3.4577E-13 |
| 7448662 | -1.9535 | HSF_2.4e | 8.2222E-12 |
| 7450945 | 1.6067 | HSF_2.4f | 2.5589E-10 |
| 7442182 | -2.5767 | HSF_2.4g | 4.8322E-13 |
| 7450628 | 1.225 | HSF_2.5a | 0.000018826 |
| 7444530 | 8.9963 | HSF_2.5c | 1.4941E-49 |
| 7443925 | -2.0251 | HSF_2.5d | 0.0005607 |
| 7443916 | 1.0996 | HSF_2.5e | 0.016347 |
| 7443558 | -0.86997 | HSF_2.5g | 0.00034052 |
| 7453047 | -2.9199 | HSF_2.5h | 1.1061E-27 |
| 7446270 | 2.683 | HSF_2.6a | 1.0021E-17 |
| 7452984 | 9.0036 | HSF_2.6b | 1.8974E-212 |
| 7452406 | -0.38804 | HSF_2.6c | 0.6679 |
| 7452405 | -2.8703 | HSF_2.6d | 6.4149E-23 |
| 7449775 | -2.1071 | HSF_2.6e | 1.8111E-15 |
| 7448929 | -2.7891 | HSF_2.7a | 6.8415E-55 |
| 7444580 | -0.3692 | HSF_2.7b | 0.53217 |
| 7445260 | 2.5137 | HSF_2.7d | 2.5504E-23 |
| 7449827 | 0.38547 | HSF_2.7e | 0.046619 |
| 7453469 | 0.90404 | HSF_2.7f | 0.18209 |
| 7449179 | -1.6898 | HSF_2.7g | 4.2049E-13 |
| 7452737 | 3.73 | HSF_2.7h | 6.2422E-77 |
| 7442164 | 11.267 | HSF_2a | 1.5396E-99 |
| 7441933 | 2.5779 | HSF_2b | 4.5133E-06 |
| 7442494 | 2.3815 | HSF_3.2b | 2.5624E-35 |
| 7450501 | 3.0033 | HSF_3.2i | 7.5439E-43 |
| 7443766 | 4.0594 | HSF_3.2j | 5.4606E-93 |
| 7447848 | 5.3508 | HSF_3.4a | 1.1975E-09 |
| 7449828 | 1.646 | HSF_3.4b | 1.2765E-16 |
| 7443817 | -0.025531 | HSF_3.4c | 0.97745 |
| 7449205 | 3.774 | HSF_3.4d | 2.3086E-37 |
| 7444930 | -0.74957 | HSF_3.5a | 0.0019437 |
| 7442692 | 0.37967 | HSF_3.5b | 0.31951 |
| 7449912 | 5.5343 | HSF_3.5c | 2.5321E-63 |
| 7447449 | -2.0288 | HSF_3.5d | 8.8725E-16 |
| 7442285 | 4.4402 | HSF_3.5e | 1.236E-43 |
| 7449952 | -3.5876 | HSF_3.5f | 6.4276E-11 |
| 7446293 | -0.31834 | HSF_3.5g | 0.38218 |
| 7446983 | 2.1289 | HSF_3.5h | 8.4216E-25 |
| 7450415 | 2.2502 | HSF_3.6a | 4.0203E-16 |
| 7448454 | 3.9232 | HSF_3.6b | 7.6462E-29 |
| 7442664 | 1.82 | HSF_3.7a | 0.027623 |
| 7450318 | -0.60818 | HSF_3.7b | 0.0051942 |
| 7449654 | 0.058743 | HSF_3.7d | 0.76581 |
| 7447884 | 2.2502 | HSF_3.7e | 6.7369E-28 |
| 7449908 | -1.1051 | HSF_3.8a | 0.00081939 |
| 7444098 | 5.0724 | HSF_3.8b | 3.5797E-80 |
| 7443097 | 6.3745 | HSF_3c | 5.9134E-81 |
| 7445707 | 0.65129 | HSF_4.2b | 0.0025617 |
| 7444191 | 5.9121 | HSF_4.2c | 6.8818E-77 |
| 7447211 | 2.6658 | HSF_4.2f | 4.3068E-21 |
| 7442309 | 0.93159 | HSF_4.2g | 0.00065056 |
| 7451370 | 4.7617 | HSF_4.7b | 8.7015E-66 |
| 7450428 | 2.7123 | HSF_4.7c | 1.1689E-32 |
| 7449826 | 4.9249 | HSF_4c | 8.4034E-124 |
| 7452998 | -0.97347 | HSF_4d | 0.0024821 |
| 7444112 | 1.867 | HSF_4e | 0.00073602 |
| 7451045 | -0.70357 | Myb1R_SHAQKYF1 | 0.00012868 |
| 7444411 | 1.4085 | Myb1R_SHAQKYF10 | 5.0462E-06 |
| 7448607 | -0.64979 | Myb1R_SHAQKYF2 | 0.56799 |
| 7452244 | 1.0779 | Myb1R_SHAQKYF3 | 4.4785E-08 |
| 7446536 | -2.0418 | Myb1R_SHAQKYF5 | 1.0639E-06 |
| 7451146 | -0.72956 | Myb1R_SHAQKYF9a | 0.0002903 |
| 7449819 | 7.0411 | Myb1R_SHAQKYF9b | 0.095196 |
| 7450547 | -1.5388 | Myb1R1 | 0.0067847 |
| 7448700 | -2.492 | Myb1R10 | 9.4488E-20 |
| 7451526 | -2.252 | Myb1R11 | 2.0634E-27 |
| 7445045 | -1.6776 | Myb1R2 | 1.4693E-13 |
| 7448550 | -0.401 | Myb1R3 | 0.23362 |
| 7445539 | -2.7161 | Myb1R4 | 1.1155E-51 |
| 7449289 | 1.7382 | Myb1R6 | 6.9228E-09 |
| 7442545 | 0.25556 | Myb1R8 | 0.66912 |
| 7443744 | 2.9194 | Myb1R9 | 1.5378E-22 |
| 7449263 | -2.6293 | Myb2R1 | 6.1124E-09 |
| 7448713 | 4.1341 | Myb2R2 | 8.6249E-84 |
| 7447935 | -0.13558 | Myb2R3 | 0.83618 |
| 7450669 | -1.6225 | Myb2R4 | 2.4439E-08 |
| 7447065 | -1.0015 | Myb2R6 | 0.025648 |
| 7445231 | 0.75868 | Myb2R7 | 0.23209 |
| 7449931 | 0.69572 | Myb2R8 | 0.01808 |
| 7442773 | -5.5411 | Myb2R9 | 4.614E-120 |
| 7448222 | 0.37896 | Myb3R1 | 0.29996 |
| 7452505 | -0.14963 | Myb3R4 | 0.91045 |
| 7444894 | -0.0095704 | Myb3R5 | 0.9761 |
| 7444004 | 5.5931 | Myb3R7 | 1.2993E-109 |
| 7442244 | -2.383 | Myb4R1a | 5.3623E-08 |
| 7442245 | -1.65 | Myb4R1b | 1.3225E-07 |
| 7449552 | 1.854 | Myb4R2 | 0.00067072 |
| 7443886 | 0.16469 | Myb4R3 | 0.5456 |
| 7445504 | 0.24764 | sigma70.1 | 0.4722 |
| 7443452 | 1.4387 | sigma70.2 | 1.282E-12 |
| 7446762 | 1.4217 | sigma70.3a | 0.00022516 |
| 7447002 | 0.10139 | sigma70.3b | 0.81548 |
| 7451383 | -0.51579 | sigma70.4 | 0.061277 |
| 7452665 | 1.6714 | sigma70.5 | 7.4362E-17 |
| 7448581 | 2.3222 | sigma70.6 | 3.9043E-26 |
| 7446212 | 3.9165 | sigma70.7 | 1.1125E-79 |
| 7442242 | -1.0755 | TAF9 | 0.000016389 |
| 7451433 | 0.56601 | TAZ1 | 0.0602 |
| 7446265 | 1.1144 | TAZ2 | 0.029906 |
| 7445864 | -2.2617 | TAZ3_PHD1 | 1.9974E-32 |
| 7443298 | -1.9244 | TAZ4_PHD2_ZZ2 | 2.9015E-25 |
| 7443299 | -0.43385 | TAZ5_PHD3_ZZ3 | 0.023665 |
| 7451434 | 1.4798 | TAZ6 | 1.5689E-10 |

**Table S6. Global distribution of the Chitin synthase B.**

Large tables that extend beyond the width of the page will be provided as separate files in an appropriate spreadsheet format (.xlsx).

**Table S7. Global distribution of the Chitinase.**

Large tables that extend beyond the width of the page will be provided as separate files in an appropriate spreadsheet format (.xlsx).

**Table S8. Global distribution of the SIT1.**

Large tables that extend beyond the width of the page will be provided as separate files in an appropriate spreadsheet format (.xlsx).

**Table S9. Global distribution of the AP2-1.**

Large tables that extend beyond the width of the page will be provided as separate files in an appropriate spreadsheet format (.xlsx).

**Table S10. Primers and sgRNA sequences used in this study.**

| Primer name | Sequences |
| --- | --- |
| OE-CinW2-F | ATCGATATCATAATCATGAAGCTCGCTCTCTTCCT |
| OE-CinW2-R | CGGGGTACCCCATCCACTGTACCATCCCTCGT |
| KO-CinW2-gRNA1-F | ACAATCCTCCGTTCGTGGAG |
| KO-CinW2-gRNA1-R | CAACTCCGGCAAATCAGGCA |
| KO-CinW2-gRNA2-F | TGGGAAGGTTCCTATGGAAG |
| KO-CinW2-gRNA2-R | CTTCCATAGGAACCTTCCCA |
| KO-CinW2-gRNA3-F | GACTCATCCAAGTCAAGCAA |
| KO-CinW2-gRNA3-R | TTGCTTGACTTGGATGAGTC |
| TpActin-like-qF | CGAAACCTATCCACGACGTT |
| TpActin-like-qR | CTCCCAATCCTGGCAATAGA |
| TpCinW2-qF | GGGAGGACACTACGATGTTG |
| TpCinW2-qR | CGAAGAGGATGAACCCTTGG |
| TpSil4-qF | GAAGTTCTGAAGCCACGACA |
| TpSil4-qR | CATCTCCCCTTTGTGAGCCT |
| TpChitin synthase B-qF | ACTATTTGCTCGGACAGAAA |
| TpChitin synthase B-qR | GTAGAAGTTTGGTAGAGAGA |
| TpChitinase-qF | TGATACTGGCAGGCACTTCG |
| TpChitinase-qR | GACCGTTGACCACGATCCAT |

**Table S11. Primers used for Y1H and Dual-Luciferase assays.**

| Primer name | Sequences |
| --- | --- |
| phis2-CinW2-F | TCACTATAGGGCGAATTCTTGGATGGAGTGGTGGATCGAGAGG |
| phis2-CinW2-R | TCGCGAACGCGTGAGCTCGGTTTCTTTTGTTCGTTTATCGTTGAGTTG |
| phis2-Chitinase-F | TCACTATAGGGCGAATTCTATTGTACAAGGGCTCACTTCTAATGATTG |
| phis2-Chitinase-R | TCGCGAACGCGTGAGCTCATAAATTAGTCCTTGTTCAAAGTGATGCGG |
| phis2-7446358-F | TCACTATAGGGCGAATTCCGTGTCAATCATGAGGCTTTGTCTCG |
| phis2-7446358-R | TCGCGAACGCGTGAGCTCGACCACAAATATAGCAACCTTCTCCC |
| AD-AP2-F | GTACCAGATTACGCTCATATGATGGCGCTAACTGTGCAACAATGG |
| AD-AP2-R | ATGCCCACCCGGGTGGAATTCCTACACAATCTCTCTGTATTTCAAACG |
| 0800-CinW2-F | GTCGACGGTATCGATAAGCTTTTGGATGGAGTGGTGGATCGAGAGG |
| 0800-CinW2-R | CGCTCTAGAACTAGTGGATCCGGTTTCTTTTGTTCGTTTATCGTTGAGTTG |
| 0800-Chitinase-F | GTCGACGGTATCGATAAGCTTTATTGTACAAGGGCTCACTTCTAATGATTG |
| 0800-Chitinase-R | CGCTCTAGAACTAGTGGATCCATAAATTAGTCCTTGTTCAAAGTGATGCGG |
| 0800-7446358-F | GTCGACGGTATCGATAAGCTTCGTGTCAATCATGAGGCTTTGTCTCG |
| 0800-7446358-R | CGCTCTAGAACTAGTGGATCCGACCACAAATATAGCAACCTTCTCCC |
| 62SK-AP2-F | TCTAGAACTAGTGGATCCATGGCGCTAACTGTGCAACAATGG |
| 62SK-AP2-R | GACGGTATCGATAAGCTTCACAATCTCTCTGTATTTCAAACG |

**Table S12. Statistical analyses.**

Large tables that extend beyond the width of the page will be provided as separate files in an appropriate spreadsheet format (.xlsx).
